# The dietary impact of the Norman Conquest: A multiproxy archaeological investigation of Oxford, UK

**DOI:** 10.1371/journal.pone.0235005

**Published:** 2020-07-06

**Authors:** Elizabeth Craig-Atkins, Ben Jervis, Lucy Cramp, Simon Hammann, Alexandra J. Nederbragt, Elizabeth Nicholson, Allie Rae Taylor, Helen Whelton, Richard Madgwick

**Affiliations:** 1 Department of Archaeology, The University of Sheffield, Sheffield, United Kingdom; 2 School of History, Archaeology and Religion, Cardiff University, Cardiff, United Kingdom; 3 Department of Anthropology and Archaeology, University of Bristol, Bristol, United Kingdom; 4 Department of Chemistry and Pharmacy, Friedrich-Alexander University Erlangen-Nürnberg, Erlangen, Germany; 5 Spokane Tribe of Indians Preservation Program, Wellpinit, WA, United States of America; 6 Organic Geochemistry Unit, School of Chemistry, University of Bristol, Bristol, United Kingdom; Universita degli Studi di Milano, ITALY

## Abstract

Archaeology has yet to capitalise on the opportunities offered by bioarchaeological approaches to examine the impact of the 11th-century AD Norman Conquest of England. This study utilises an integrated multiproxy analytical approach to identify and explain changes and continuities in diet and foodways between the 10th and 13th centuries in the city of Oxford, UK. The integration of organic residue analysis of ceramics, carbon (δ^13^C) and nitrogen (δ^15^N) isotope analysis of human and animal bones, incremental analysis of δ^13^C and δ^15^N from human tooth dentine and palaeopathological analysis of human skeletal remains has revealed a broad pattern of increasing intensification and marketisation across various areas of economic practice, with a much lesser and more short-term impact of the Conquest on everyday lifestyles than is suggested by documentary sources. Nonetheless, isotope data indicate short-term periods of instability, particularly food insecurity, did impact individuals. Evidence of preferences for certain foodstuffs and cooking techniques documented among the elite classes were also observed among lower-status townspeople, suggesting that Anglo-Norman fashions could be adopted across the social spectrum. This study demonstrates the potential for future archaeological research to generate more nuanced understanding of the cultural impact of the Norman Conquest of England, while showcasing a method which can be used to elucidate the undocumented, everyday implications of other large-scale political events on non-elites.

## Introduction

The Norman Conquest of England in AD 1066 led to profound political and economic change. In the short term, the advance of the Norman army devastated the countryside, disrupting local political and economic infrastructures and causing starvation in areas of northern England [1]. Longer-term changes to urban landscapes, including the construction of castles, cemented Norman control over existing centres of power and commerce [2,3]. While the impact of the Conquest on power dynamics and the political elite is evident in both the historical and archaeological record, its effect on the everyday lives of the English population remains poorly understood [4]. This paper utilises an integrated multiproxy analytical approach to the study of diet as a means of examining the impact of the Norman Conquest of England on non-elite households, and in doing so demonstrates the potential of such an approach for illuminating the complexity of key periods of political change in the past.

The study of diet enables exploration of the impact of political change on everyday life through its illumination of the provisioning, marketing, selection and preparation of foodstuffs. Food and drink culture is highly sensitive to social context and is utilised as a means of both rationalising and expressing differences within and between groups [5–7]. Previous analyses of diet in England during the 11th-century Norman Conquest have focussed on changes in food culture brought about by French cultural influences. These include increased consumption of domesticated pigs and wild species, as well as changes in cooking practices, such as a greater frequency of roasting and new methods of slow cooking, which have been shown to be most marked among the political and economic elite [8,9]. These changes can be set against developments in food culture, which characterise the early centuries of the late medieval period, such as the increasing consumption of fish, which have been linked to the growth of urban centres and tighter observance of religious proscriptions [8,10], and a shift away from dairying towards meat production at rural ‘producer’ sites [4].

This project utilised a multiproxy methodology to examine the long- and short-term impact of the Norman Conquest on diet. Multiple analytical techniques were applied to three types of archaeological evidence–ceramics, animal remains and human remains–from excavations across the city of Oxford, to examine dietary change between the 10th and 13th centuries. The approach capitalised on archaeological-science techniques, integrating organic residue analysis of ceramics, carbon (δ^13^C) and nitrogen (δ^15^N) isotope analysis of human and animal bones, incremental analysis of δ^13^C and δ^15^N in human tooth dentine and palaeopathological analysis of human remains. This multiproxy approach enabled the generation of high-resolution and multiscalar data concerning the impact of the 11th-century Conquest on foodways at both individual and community levels, facilitating evaluation of key questions concerning continuity of food supply, production and marketing, and nutritional status of the populace in one locale. It also focussed on developments in food culture and cuisine, in particular establishing whether the changes identified in elite foodways could be observed in a non-elite urban context.

## Materials and methods

**Fig 1:**
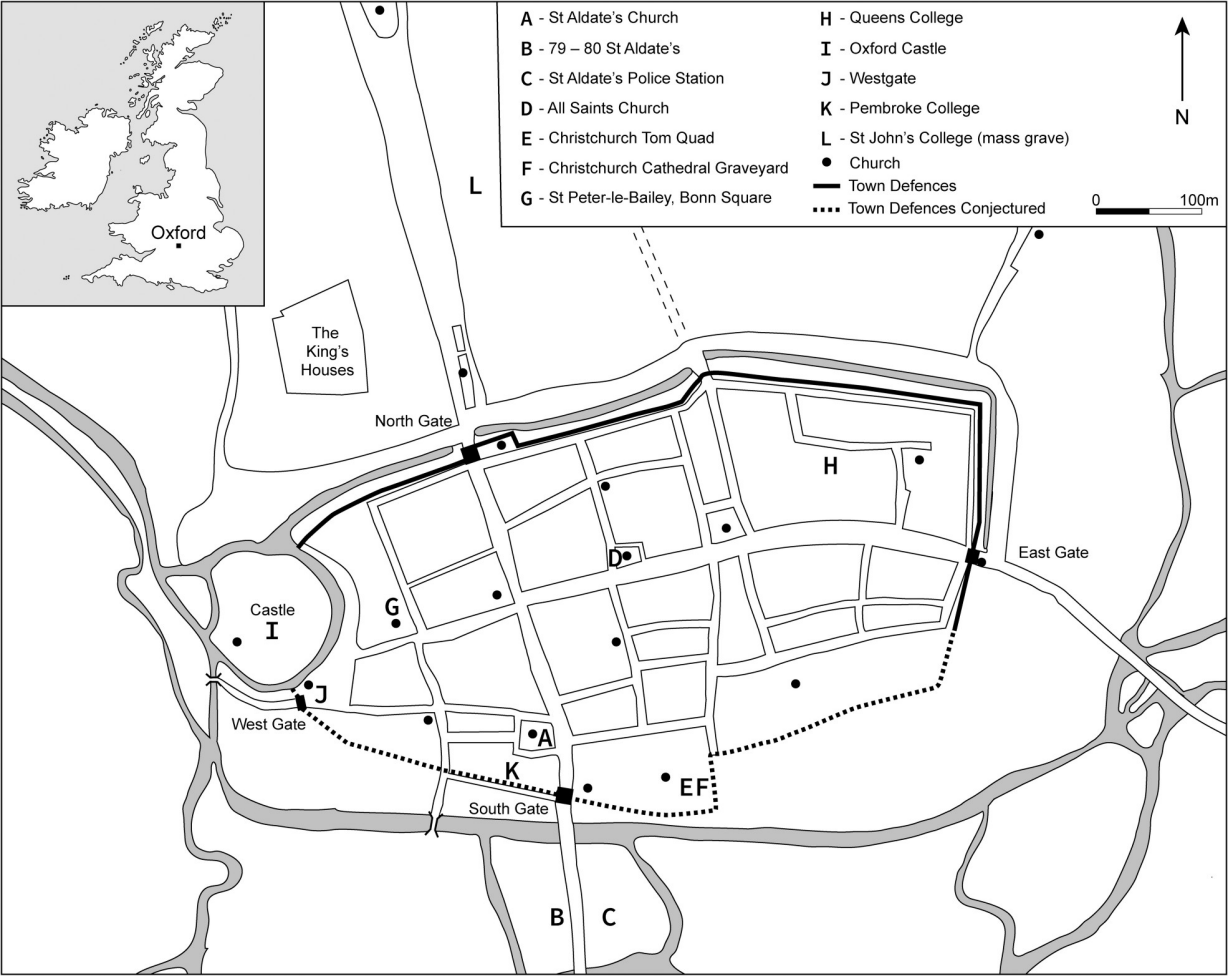
Plan of medieval Oxford showing the sites included in the present study. St John’s College (L) is included in this study as comparative data only.

A group of archaeological sites from Oxford and its environs were selected for analysis based on quantity and quality of their archaeological archives and the accessibility, quantity and preservation of pottery, animal remains and human remains for analysis [11–23]. Reliable dating evidence was necessary if a suitably precise chronology was to be developed. The city of Oxford benefits from both a well-understood archaeological ceramic sequence [24] and numerous sites with chronologies supported by radiocarbon dates, which could be targeted for analysis. Where possible, data were separated into three chronological groups: pre-Conquest (c. 10th century), 11th-century and post-Conquest (c. 12th century) to reflect our aspiration to understand change as a continuous process across the Conquest period, in contrast to a single event marked by the historical threshold of AD 1066. Nevertheless, in some cases the resolution of dating evidence was insufficient to define three periods, particularly where stratigraphic or pottery dates were not available to refine the relatively broad radiocarbon chronology, or the availability of samples necessitated the lumping of data to enable meaningful discussion. In total, 12 archaeological sites were identified, and samples selected from across these sites to facilitate each of the analytical approaches (Fig 1, Table 1). The archaeological materials were archived with Oxfordshire Museum Resource Centre, Standlake, Oxfordshire and accessed for analysis at this facility.

**Table 1:** Pottery, animal bone and human bone samples included in the present study.

Site	Pottery residue analysis samples (n)		Animal bone stable isotope samples (n)		Human bone stable isotope samples (total osteological sample) (n)		
	Pre-Conquest	Post-Conquest	Pre-Conquest	Post-Conquest	Pre-Conquest	11th Century	Post-Conquest
79–80 St Aldate's	3	-					
St Aldate's Church	11	8			(10)	-	-
St Aldate’s Police Station^a^			-	30			
All Saints’ Church			15	-	-	9 (19)	3 (61)
St Peter-le-Bailey, Bonn Square					-	-	(111)
Christ Church Cathedral					6 (36)	-	-
Christ Church Cloister					(17)	-	-
Christ Church Tom Quad					-	-	(4)
Oxford Castle			15	-	1 (2)	4 (11)	-
Queen’s College	8	8					
Pembroke College	-	3					
Westgate					-	-	13 (13)
***Total***	*22*	*19*	*30*	*30*	*7 (65)*	*13 (30)*	*16 (189)*

a. Includes the Police Station and Land Adjacent to the Police Station excavations.

The materials and methods are summarised briefly here with more detail provided in S1 Data. No permits were required for the described study, which complied with all relevant regulations.

Forty-one samples of pottery from jars (cooking pots) that showed evidence of being used in cooking were selected for organic residue analysis to generate direct evidence of foodstuffs stored, prepared and served in the vessels. Bone samples from 60 animals, equally split between pigs, caprines and cattle, were obtained for isotope analysis of δ^13^C and δ^15^N. These data revealed information about the diets of these individuals, thus provided direct evidence of livestock management strategies and a baseline from which to reconstruct human diets. Samples were selected from among the remains of 248 human individuals for stable isotope analysis of bone collagen (n = 38), stable isotope analysis of incremental dentine (n = 9) and osteological assessment of both dental pathology and non-specific markers of physiological stress (n = 235). These data provided direct evidence of diet averaged over several years prior to death, high-resolution insights into dietary change during childhood at an individual level and an insight into skeletal health and dietary pathologies affecting the dentition, respectively.

In synergy, organic residue analysis of ceramics, δ^13^C and δ^15^N isotope analysis of human and animal bones, incremental δ^13^C and δ^15^N isotope analysis of human dentine and osteological analysis of human remains have the potential to support nuanced interpretations of dietary variability at a temporal resolution suitable for exploring the impact of the Norman Conquest on foodways.

## Results and discussion

To examine the impact of the Conquest on foodways in Oxford, analysis and discussion of the data are integrated in the following sections under thematic headings associated with diet: food preparation, consumption and cuisine; animal husbandry; and food supply and dietary change.

### Food preparation, consumption and cuisine

Absorbed residues in pottery vessels provide direct evidence of food preparation and consumption practices by characterising the foodstuffs stored and cooked in pots. In total, 68.3% (28/41) of the sampled potsherds contained lipid concentrations considered significant (> 5 μg g^-1^ sherd; S1 Table).

**Fig 2:**
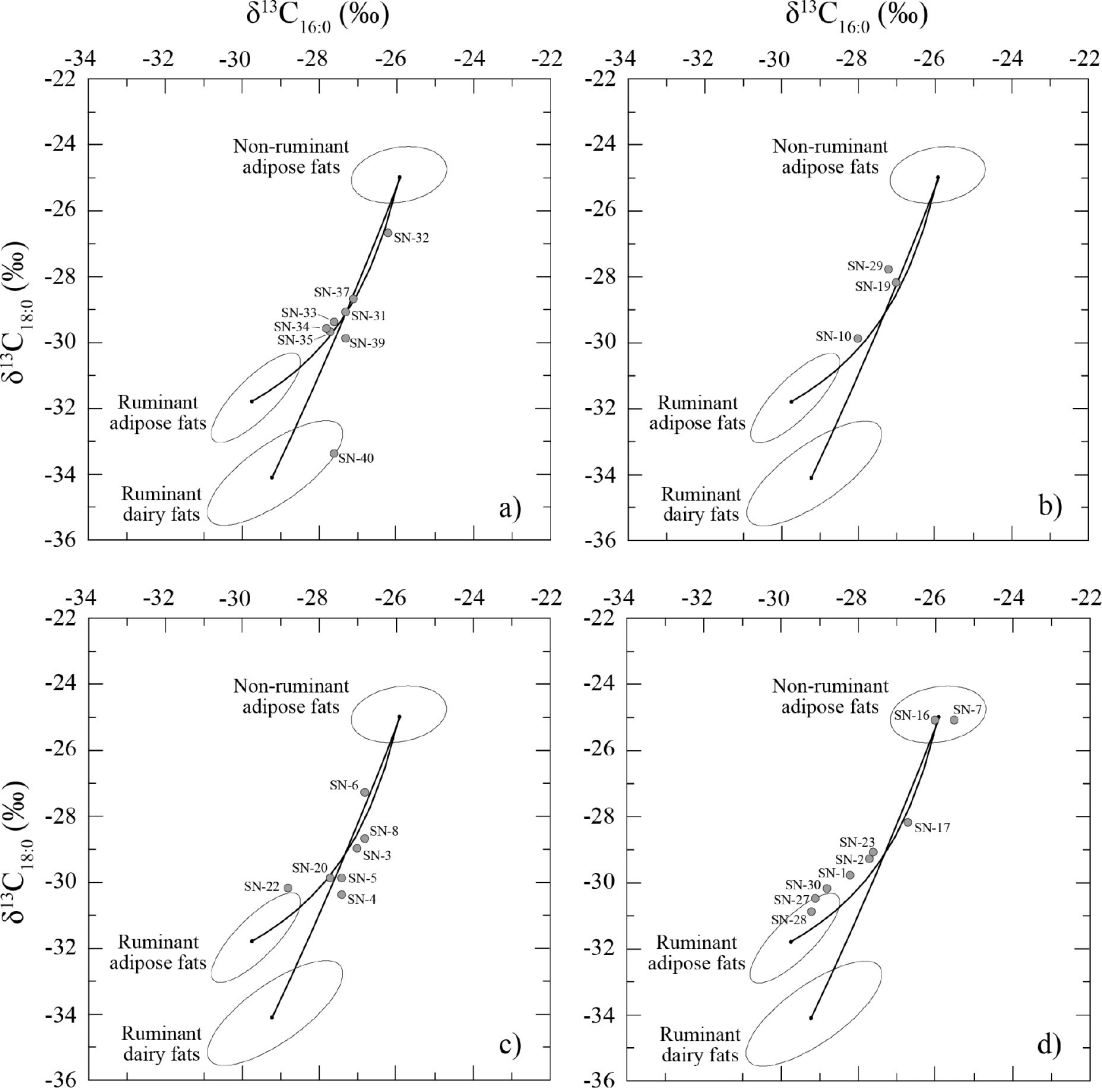
Scatter plots showing the δ^13^C values of C_16:0_ and C_18:0_ fatty acids prepared from total lipid extracts of the pottery. (A) mixing plot and (C) big delta plot of extracts from pre-Conquest sherds (Late Saxon Shelly ware c. AD 900–1050 and St Neots ware c. AD 925–1050); (B) mixing plot and (D) big delta plot of extracts from post-Conquest sherds (Cotswold ware c. AD 1050–1250, and Oxford ware c. AD 1050–1250). The values of modern reference fats are represented by confidence ellipses (1 standard deviation). All δ^13^C values obtained for modern reference animal fats have been adjusted for the post-Industrial Revolution effects of fossil fuel burning, by the addition of 1.2‰ [25]. Lines connecting the ellipses represent theoretical δ^13^C values obtained through the mixing of these fats.

**Fig 3:**
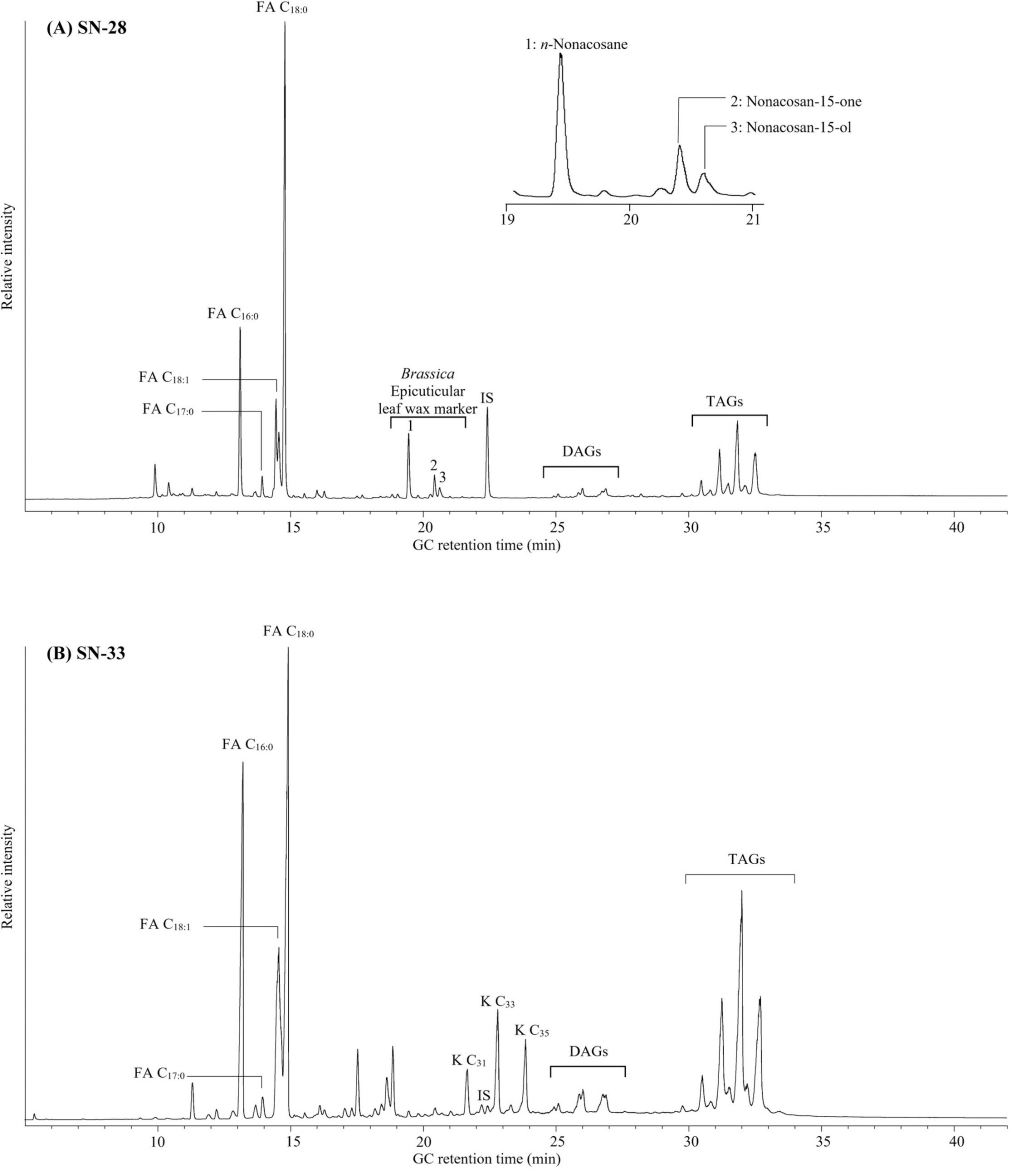
A partial high temperature gas chromatograms of degraded animal residues. (A) from sherd SN-28, also showing presence of *Brassica* biomarkers. FA C*x*:*y–*free fatty acid with carbon chain length *x* and *y* degree of unsaturation. DAGs–diacylglycerol, TAGs–triacylglycerol. IS–internal standard, C_34_ *n*-alkane. (B) From sherd SN-33 K C*x*–mid-chain ketone with carbon chain length *x*.

Saturated free fatty acids, dominated by the C_16:0_ and C_18:0_ homologues, were present in the twenty-eight viable lipid extracts and confirmed the presence of degraded animal fats (Figs 2, 3A and 3B). Most vessels were used for animal product processing and there was abundant evidence, based upon the single compound stable carbon isotope determinations, for use of ceramic vessels of all periods for meat from ruminants, such as cattle, sheep and/or goat. However, the organic residues suggest variation in the use of vessels that might tentatively be associated with change across the Conquest period. Evidence of dairy fats was scarce but exclusive to pre-Conquest sherds: one of Late Saxon Shelly Ware from St Aldate’s (SN-40) and one of St Neot’s Ware from Queen’s College (SN-4). In contrast, residues dominated by pork and/or chicken fats were only obtained from two post-Conquest Oxford Ware vessels–the latest stratigraphically-dated contexts in this study (SN-7 –AD 1200–1300; SN-16 –AD 1175–1250). Mixed ruminant and non-ruminant adipose residues were, however, found in both pre- and post-Conquest sherds. In sum, the organic residue evidence indicates most vessels were used for cooking beef, lamb and/or goat meat, but also suggests the possibility of a shift in the post-Conquest pottery away from dairy and towards the predominant use of some pots for pork and/or chicken processing.

Results of the targeted analysis of cereal biomarkers detected traces of plant sterols and stanols alongside long-chain *n-*alcohols in the majority of extracts, suggesting pots of both periods were used for processing of plants (S1 Table). Although specific enrichment by solid phase extraction from the total lipid extracts and sensitive analysis by GC-QTOF MS was performed, alkylresorcinols–biomarkers specific to cereals–were not detected in any of the 41 sherd extracts. In this context, an absence of archaeological traces of alkylresorcinols cannot be interpreted as a lack of cereal processing since it is known that burial under oxic conditions is likely to promote rapid degradation of these compounds [26]. Moreover, oats contain only very low traces of alkyresorcinols [27] and would not be detectable using these biomarkers.

Evidence of compounds typical of Brassica (cabbage-type) epicuticular leaf wax, such as nonacosane, nonacosa-15-one and nonacosan-15-ol [28] was identified in six potsherds from both pre- and post-Conquest periods (SN-5, 6, 10, 23, 27 and 28; Fig 3A). All sherds with evidence of processing of plants from the *Brassica* genus, regardless of date, site or pottery form, had also been used for ruminant meat processing, thus evidencing the frequent use of key ingredients of beef, lamb and/or goat and leafy vegetables across the 10th-13th centuries.

Evidence can also be drawn from the lipid analysis for the heating of the vessels and therefore practices of food preparation. Mid-chain ketones in the range of C_31_ to C_35_ were identified in seven sherds of Late Saxon Shelly Ware (SN-31, 32, 33, 34, 35, 37 and 39; Fig 3B). Based on the range and pattern of homologues observed (C_31_ –C_35_) in these particular sherds, the ketones present in the lipid extracts were formed through condensation of two fatty acids temperatures exceeding 300°C, catalyzed by the clay surface [29], which provides evidence that these vessels have been heated to high temperatures.

In combination, the results from the organic residue analyses indicate a degree of long-term consistency in consumption of meat from ruminant animals and leafy vegetables across the Conquest period. Presence of dairy products was rare, but distinctive to pre-Conquest assemblages and the only examples with fatty deposits exclusively from non-ruminant adipose, most likely pork and/or chicken, were of later post-Conquest date. Sample sizes were small and therefore these data must be interpreted with caution. Nonetheless, our findings from non-elite urban contexts are consistent with the previously observed increase in the consumption of non-ruminant protein in elite households alongside an apparent decrease in the cooking or storage of milk-based fats [8].

Our findings may be compared with the only other study of residues from the Conquest period, of the rural manorial complex of West Cotton, Raunds, Northamptonshire [30, 31]. Here, organic residues from 73 vessels (123 sherds) spanning the pre-Conquest (AD 950–1150), manorial (AD 1100–1250) and manor/hamlet (AD 1250–1400) phases revealed evidence for preparation of predominantly ruminant products, alongside leafy vegetables including biomarkers for the *Brassica* and *Allium* genera. Evidence for predominantly non-ruminant product processing was absent from the six pre-Conquest sherds analysed from this site, but it was identified in 2/10 pottery residues from the AD 1100–1250 assemblage, similarly suggesting an increasing prevalence of pork or chicken in the diet alongside continuing consumption of ruminant animals. The residues from West Cotton also support long term continuity in consumption of leafy vegetables. In contrast with our findings, however, dairy products were similarly prevalent in sherds from both the pre-and post-Conquest periods, with no apparent decline. One possibility is that this reflects differences in access to processed *versus* non-processed dairy products in medieval urban and rural settings, leading to lower visibility of dairy products in pots from the former. It may also reflect different dietary patterns or specialisation of vessel forms, the latter hinted at by the Dunne et al. study.

Archaeobotanical evidence for medieval Oxford enables expansion of the discussion of processing and consumption of plant-based foods. Plant, invertebrate and pollen samples from waterlogged sediments at St Aldate’s produced radiocarbon dates spanning the Conquest period and a charred grain deposit from All Saints’ Church has been dated to the 10th century. These reveal a diverse range of potentially-cultivated food resources available to the people of Oxford, which include free-threshing wheat, rye, field/broad bean, celery, apple, blackberry, *Prunus* sp. (plum, damson, sloe, etc.) and the herb Summer Savoury (*Satureja hortensis*), which was likely used to flavour meat dishes [32–33]. Pollen from faecal material in a cesspit of c. 13th century date indicated a diet including a variety of cereals [32]. The large deposit of charred free-threshing wheat spread across 10th-century layers at All Saints’ Church also supports the importance of cereals, in this case indicating the storage (and disastrous destruction by fire) of large quantities of very pure threshed and fully-cleaned wheat within the town [33].

New cooking practices have also been reported in Anglo-Norman elite culture, including a trend towards slower cooking, use of different cooking vessels and an increase in practices such as roasting [8]. Variations in the position and type of sooty deposits on pottery vessels fromƒ late Saxon and Anglo-Norman Southampton have also been interpreted as evidence of change in cooking practice, in this case suspension of a vessel above the fire in post-Conquest kitchens instead of placement directly in the embers [34]. However, the impact of the Conquest is not the only explanation that has been advanced for variation in cooking practices during the 10th-13th centuries in Oxford; traditions linked to ethnic groups have also been postulated [35]. In this model, Late Saxon Shelly Ware was in regular use at the centre of the town by the Saxon inhabitants, whereas St Neots Ware, which had no evidence of ketones in our study, was found in peripheral contexts and used by settlers from the Danelaw (the area of northern and central England under Danish control).

### Animal husbandry

**Fig 4:**
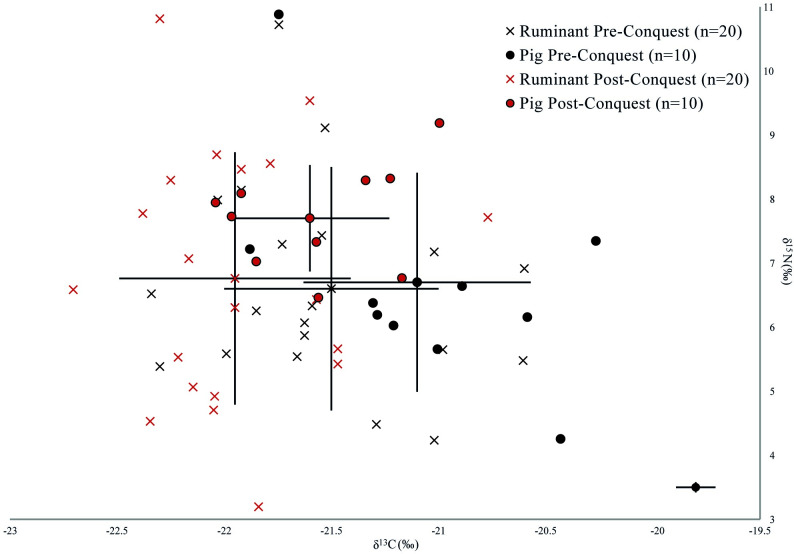
δ^13^C and δ^15^N isotope data from animal bone collagen organised by phase. Error bars represent one standard deviation and analytical error is displayed at the bottom right of the graph.

Results from faunal stable isotope analyses offer an insight into husbandry practices and also provide a baseline for interpretations of protein sources in human diets. The data obtained from stable isotope analyses of faunal bone from Oxford are summarised in Table 2 and Fig 4, and presented in detail in S2 Table.

**Table 2:** Mean and standard deviation figures for δ^13^C and δ^15^N values for animal bone.

		n	δ^13^C	SD	δ^15^N	SD
Pre-Conquest	Sheep/Goat	10	-21.3	0.5	6.6	1.9
	Pig	10	-21.1	0.5	6.7	1.7
	Cattle	10	-21.8	0.4	6.6	1.2
Post-Conquest	Sheep/Goat	10	-22.0	0.5	6.6	2.0
	Pig	10	-21.6	0.4	7.7	0.8
	Cattle	10	-21.9	0.3	6.7	1.9

Each taxon shows great diversity considering the small dataset, especially in terms of δ^15^N values, but there are distinct patterns of variation between species and across the Conquest. Pigs show substantially higher δ^15^N values and more negative δ^13^C values in the post-Conquest period than the pre-Conquest period. Post-Conquest pigs also show a different signature to other fauna of the same period, as they have much more consistent δ^15^N values across the sample and notably higher δ^13^C values than caprines. In contrast, pre-Conquest pigs have comparable δ^15^N values to caprines and cattle. These data suggest that pre-Conquest pigs were largely herbivorous, but with a varied diet suggesting diverse management regimes, and with animals potentially sourced from a wide hinterland with diverse baseline ecosystem isotope signals [36, 37]. That some pigs had higher δ^13^C values would be consistent with the exploitation of seasonal pannage [36, 38, 39]. The post-Conquest pig δ^13^C values exhibit a more consistent omnivorous signature with a restricted range of values, suggesting that both a more structured management strategy was adhered to and larger quantities of animal protein were utilised as fodder. It is possible plant fodder from manured landscapes also contributed to these elevated values. However, this is considered unlikely, as it would be expected to be more clearly visible in cattle and sheep. When considered alongside archaeological evidence for increasing urbanism in Oxford following the Conquest [13], this might indicate that changes in husbandry resulted in animals being more tightly managed within the confines of the city as sty pigs, being fed on waste from the growing population.

While the pre- and post-Conquest cattle show very little difference, pre-Conquest caprines have higher δ^13^C values. This is likely to relate to variable origins, with sheep being brought from different areas of the hinterland, which have varied landscape baseline values [40]. However, an increased use of leaf fodder [41] in the post-Conquest period cannot be entirely excluded. In combination, the intra-taxonomic variability suggests that both caprines and cattle were raised on a range of pastureland, both manured and unmanured, in different areas of the surrounding landscape, and that this practice remained consistent over time. Limited change in management of cattle and sheep across the Conquest has also been observed in previous studies [4].

Past research on early medieval animal husbandry in Oxford has focussed on the application of standard zooarchaeological techniques, and these data are complementary to those generated here from stable isotope analyses. Between the 9th and 12th centuries cattle account for the majority of animal remains by fragment count, with their relative importance dropping in the 10th century in relation to an increase in the presence of sheep/goat and then in the 11th century in relation to an increasing presence of pig, which rise from representing 15% of animal remains in 9th-century deposits to 23% in 11th-century deposits [42]. Thus, zooarchaeological data compiled from multiple sites in Oxford both mirror national trends in animal husbandry and consumption identified by Sykes [8], thereby contextualising our own observations, which suggest specific changes in pig husbandry. Demographic profiles of sheep from St Aldate’s also suggest change across the 10th-11th centuries. The later assemblage has more varied demography compared to the earlier ones, suggesting a shift from a wool-driven husbandry regime (whereby most animals were shorn twice before slaughter for meat) towards the breeding of sheep specifically for the urban meat and cloth markets [42]. Analysis of bone fragmentation suggests the removal of larger bone waste to the periphery of the town which, while limiting potential for intra-site analysis [42], may also indicate more systematic management of food waste, which would be entirely consistent with the aforementioned evidence from both stable isotopic and zooarchaeological sources for closer management of all aspects of livestock husbandry in the urban environment from the 11th century.

### Food supply and dietary change

**Fig 5:**
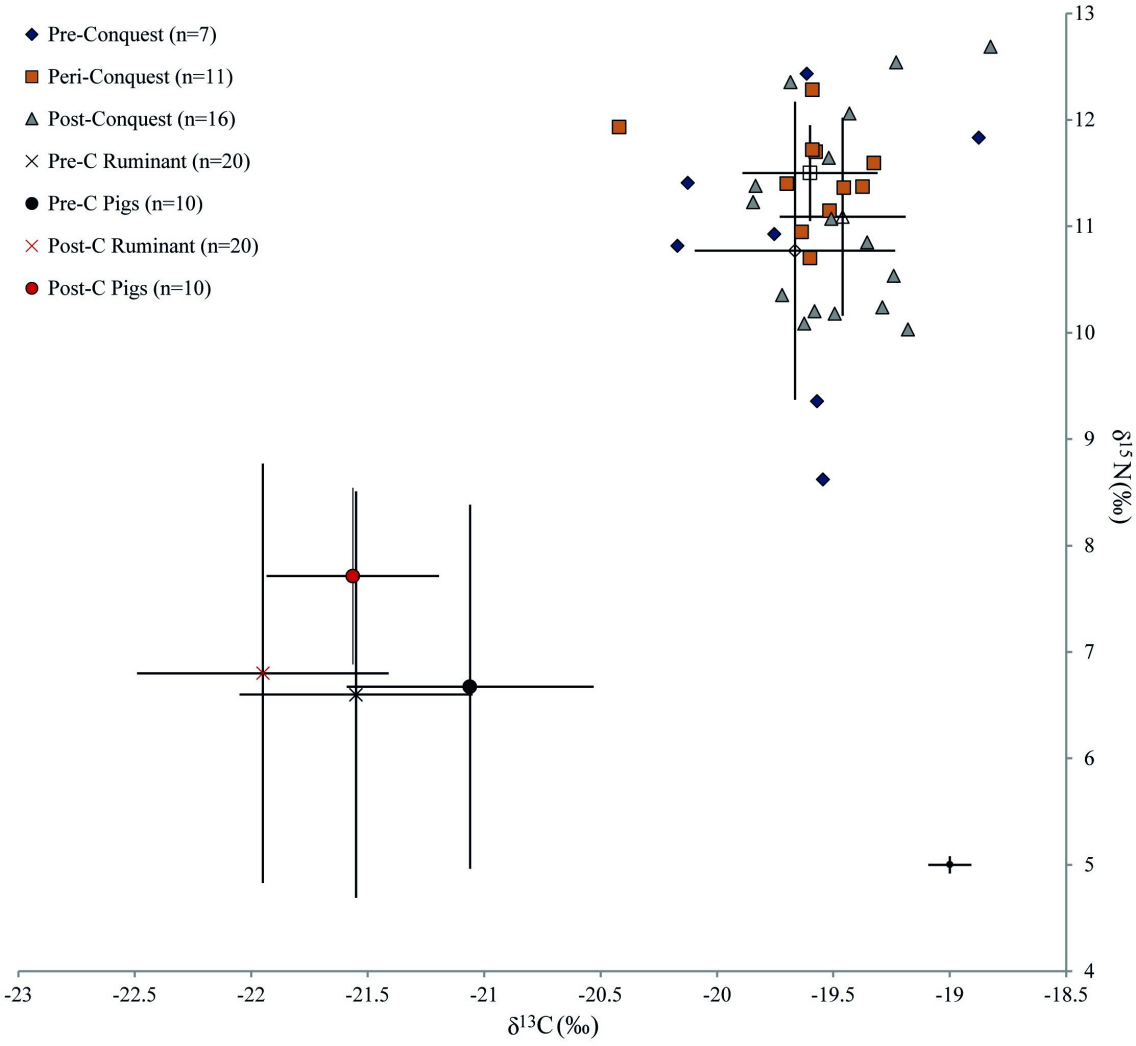
δ^13^C and δ^15^N values from animal and human bone collagen organised by phase. Error bars represent one standard deviation and analytical error is displayed at the bottom right of the graph.

The data obtained from bulk stable isotope analysis of human remains from Oxford are summarised in Table 3 and Fig 5, and presented in more detail in S3 Table.

**Table 3:** Mean and standard deviation of δ^13^C and δ^15^N for human bone.

	n	δ^13^C	SD	δ^15^N	SD
Pre-Conquest	7	-19.7	0.4	10.8	1.3
11th Century	11	-19.6	0.3	11.5	0.9
Post-Conquest	16	-19.5	0.2	11.1	0.8

The most noteworthy pattern in comparing the phased human samples is the reduction in diversity of isotope values seen from the 11th century onwards. While the standard deviations of δ^13^C values reduce consistently over the period, the standard deviation for δ^15^N values is notably higher in the pre-Conquest individuals than in both the post-Conquest and the transitional. These general trends are consistent with increased control of market supply by the Norman administration [43], which is supported by evidence for the granting of market charters in the 12th century (e.g. at Woodstock in 1154) and the referencing of established market centres in new regulatory documents (e.g. Banbury, a long-established ecclesiastical centre, was formally granted a market in 1155/6) [44]. The post-Conquest period also saw changes in rural administration, for example in Bullingdon Hundred, west of Oxford, the majority of manors came into new Norman lordship (albeit sometimes with Anglo-Saxon or Danish tenants), becoming possessions of important allies of William I [45]. Increased urbanism, a more developed market system and changes in the administration of surrounding rural areas create a context in which it is plausible that post-Conquest communities had a more homogenous diet, with a stable supply of similar produce from Oxford's hinterland, which was being more intensively managed for profit. In addition, standardised practices surrounding agricultural intensification may have resulted in local landscape baselines being homogenised, and these values being transferred up the food chain. Moreover, the results may reflect personal choice in dietary practice, for example trends in cuisine that favoured greater consumption of pork. If, as zooarchaeological evidence suggests, pig consumption increased markedly after the Conquest [8], then the reduced variability in human values may relate to greater consumption of more homogenous protein sources, potentially pork, supplemented by marine fish in some instances.

The 11^th^-century δ^15^N value mean is 0.7‰ higher than the pre-Conquest mean, but the post-Conquest mean is only 0.3‰ higher. This slight difference might hint at the consumption of more animal protein, however, as the mean post-Conquest pig δ^15^N value is 1.0‰ higher than the pre-Conquest period, it could also reflect the consumption of proportionally more pork (with higher baseline δ^15^N values) over time. The difference in human δ^13^C values is negligible but shows a slight increase through time: only one outlying pre-Conquest individual has a δ^13^C value higher than -19.5‰, whereas nine peri- and post-Conquest individuals have higher values. Although the mean differences are small, this casts some doubt over the argument for increased pork consumption, as pigs have slightly lower δ^13^C values in the post-Conquest period. Some individuals have δ^13^C values far higher than would be expected for a diet high in pork (up to 2.8‰ higher than the pig mean). Therefore, it is plausible that some marine input in the diet resulted in these higher δ^13^C values, especially for two individuals with values above -19.0‰. It is notable that individuals with particularly low δ^13^C and δ^15^N values (below c. -19.5‰ for δ^13^C and 10.5‰ for δ^15^N) are absent from the post-Conquest sample, a pattern that fits with increased access to and/or increased inclusion of non-ruminant protein and potentially some fish into the diets in the post-Conquest phase. However, the two lowest δ^15^N values are from the 11th century, suggesting variability in food supply during the transition. The consumption of freshwater fish, with the Thames being a readily accessible source, is considered unlikely due to the low δ^13^C values from freshwater fish from Roman sites in Oxford [46]. The intensification of agricultural production, however, by heavier manuring resulting in a higher nitrogen isotope baseline for consumed cereals [47], may have contributed to the higher δ^15^N values rather than pork consumption alone.

**Fig 6:**
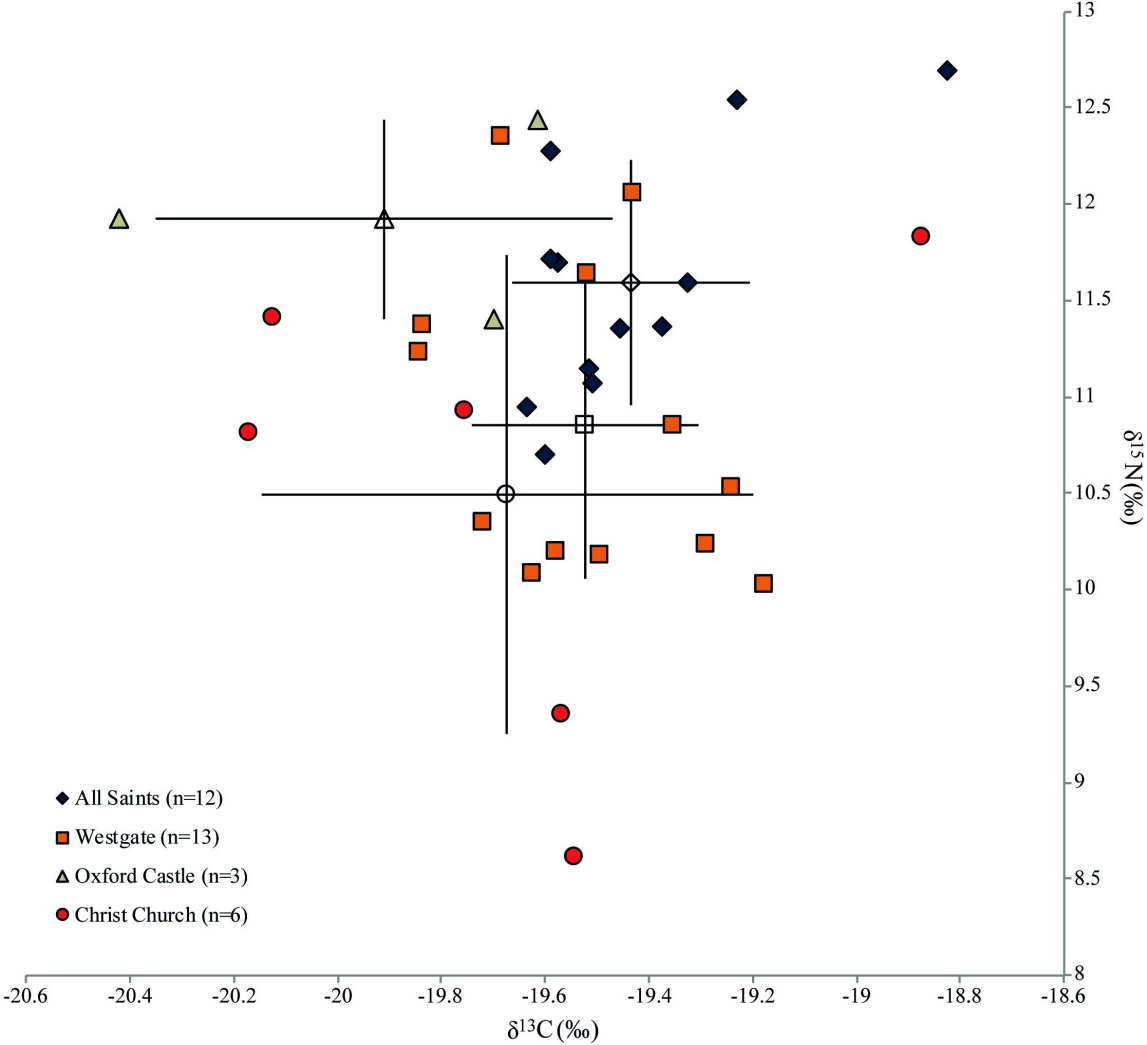
δ^13^C and δ^15^N values from human bone collagen organised by site. Error bars represent one standard deviation.

The principal aim of the isotope analysis was to assess differences in food supply and dietary practice across the period of the Norman Conquest. However, it is necessary to consider the potentially confounding effect of inter-site differences arising from the different sectors of medieval society that these cemeteries served (Fig 6). Samples sizes were very small when broken down by site, nonetheless some tentative yet interesting patterns can be noted. It might be expected, for example, that Christ Church's monastic community observed religious proscriptions against meat, and so consumed more fish (and poultry) than individuals interred in the other cemeteries. However, this appears not to have been the case, as Christ Church has the lowest mean δ^15^N and δ^13^C values compared to the other sites. The three individuals from Oxford Castle may have had a higher social status, and therefore a diet richer in meat and fish [48, 49] but in this case, as the mean δ^15^N value is highest, but the δ^13^C value is lowest, marine dietary input must have been negligible in this small sample of individuals. Two clusters of isotope values are apparent in the Westgate data, but as this material derived from a disturbed context, interpretative potential is limited. As with the interpretation of pottery residues above, some consideration must also be given to the possibility that pre-Conquest variability in diet could be linked to the ethnically-mixed composition of Oxford’s population. The higher δ^15^N values could be explained on ethnic grounds, with Danes having elevated δ^15^N values, as has been tentatively suggested in another study on remains from Oxford Castle [50]. However, while the higher variability within our pre-Conquest sample could in part be explained by ethnic variability in diet, the absence of any distinctive site-based clustering implies that, if this were the case, a mix of individuals from different ethnic groups was buried in the same locales. Only 16 individuals could be confidently assigned a sex, and the only weak observable pattern was the greater range in δ^15^N values in males (n = 9, 8.6‰-12.5‰), compared to females (n = 7, 10.9‰-11.9‰).

In sum, the isotope data from human bone collagen were highly variable, precluding a straightforward interpretation of their causative factors. However, a range of possible temporal trends in the consumption of different foodstuffs have been highlighted, suggesting increasing homogeneity in diets that can be explained by greater consistency in animal husbandry practices, by dietary choices made by individuals or a combination of both.

**Fig 7:**
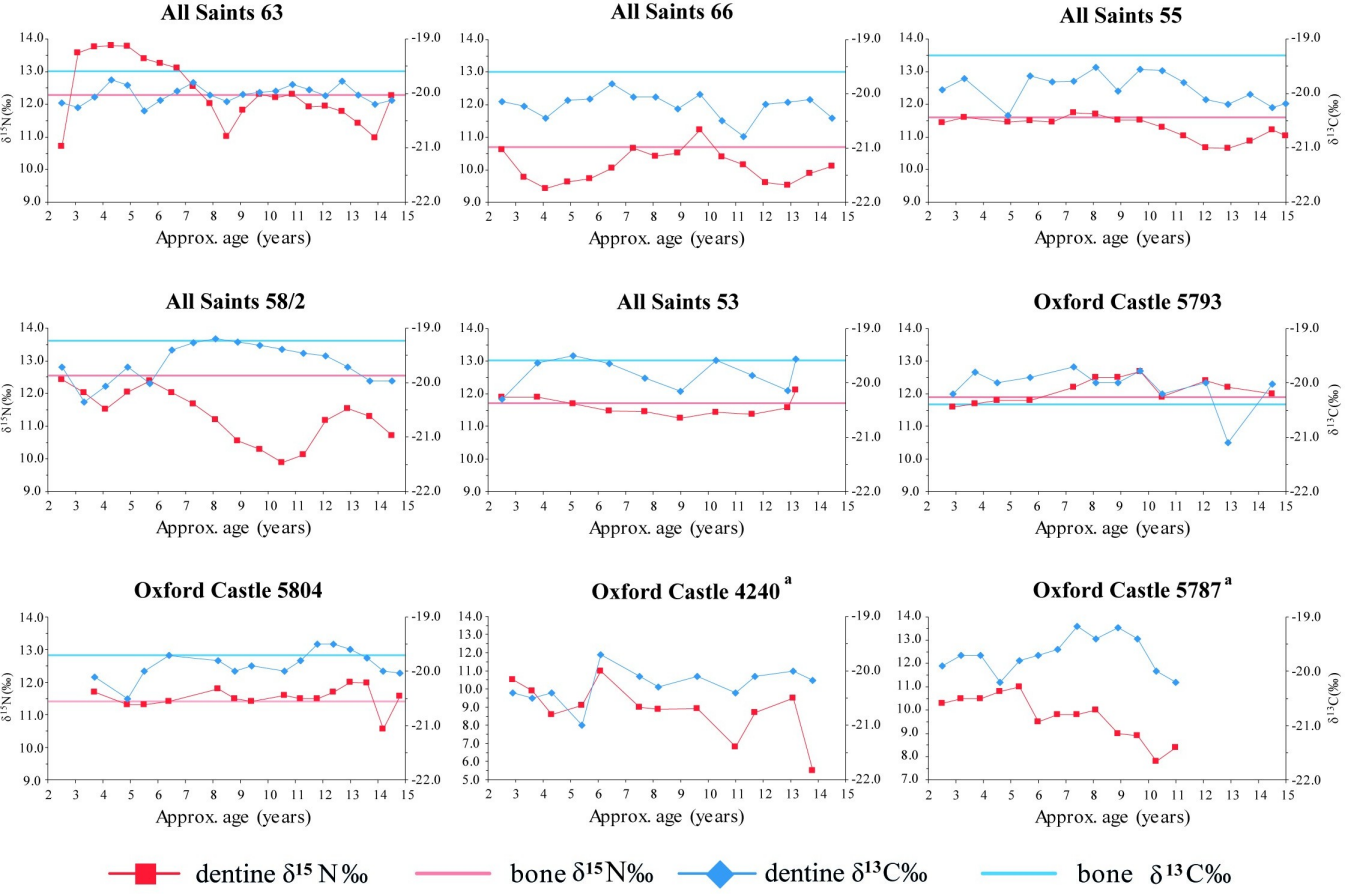
Incremental dentine δ^15^N and δ^13^C value profiles for individuals from All Saints (AST) and Oxford Castle (OXC).

Incremental δ^13^C and δ^15^N analysis was undertaken on a limited number of individuals to provide high resolution biographical data. This provides an important complementary approach to the long-term, later life trends evidenced by bone stable isotopes, not only in terms of temporal resolution, but also in showing patterns of consumption during earlier life. The analysis of second molars provides temporal resolution at the sub-annual scale for the period from approximately 2.5 to 14 years [51] to investigate dietary practice, physiological stress and food source stability. Summary incremental stable isotope data are summarised in Fig 7, and presented in more detail in S4 Table and S5 Table. δ^15^N values are more sensitive to short term change than δ^13^C values [52] and therefore it is unsurprising that the δ^13^C values (1SD ≤ 0.4) from the incremental sample were more stable than δ^15^N values (1SD 0.3 to 1.5) for all individuals. The following discussion utilises these data to examine overall variability of δ^13^C and δ^15^N values across developmental life, to consider differences in early vs later life diets (by comparing with bulk data presented above), and to evaluate the influence of factors such as physiological stress episodes and weaning patterns.

The majority of individuals showed remarkably little variation across developmental life (All Saints 53, 55, 66, Oxford Castle 5793, 5804) with the remainder showing some variation suggestive of fluctuations in diet and/or physiological stress (All Saints 63, 58–2, Oxford Castle 4240, 5787). The three most stable profiles (All Saints 53, 55, Oxford Castle 5793) all had higher δ^15^N values in both mean dentine and bone collagen than the mean animal bone value for the Conquest period, suggesting they had a substantial animal protein component in the diet. Of these, All Saints 53 (male, 17–25 years) is remarkable in having a range of only 0.8‰ for both δ^15^N and δ^13^C values with absolute values consistent with bone collagen (later life) values, indicating a high level of stability in diet across the entire life course.

Other individuals with broad stability across their profiles experienced minor or short-term perturbations in diet. Moderate variation across the profile (1.8‰ in δ^15^N, 1.0‰ in δ^13^C) with limited differences between adjacent increments (≤0.8‰) and covariation in δ^15^N and δ^13^C values suggests All Saints 66 (male, 25–35 years) experienced fluctuation in food sources rather than in physiological stress, which is associated with opposing covariance in these two isotopes [53, 54]. The absence of palaeopathological signs of physiological stress in their skeleton is consistent with this conclusion. Oxford Castle 5793 (female, 25–35 years) had a marked dip of 1.1‰ in δ^13^C values between the ages of around 12 and 13, which also suggests short-term change in prevailing food sources, although evidence of hypoplastic defects in their teeth also suggest periods of physiological stress during childhood [55]. Erratic variation in the profile of Oxford Castle 5804 (adult female), is more difficult to interpret. At around age 13 there is a 1.4‰ drop in δ^15^N values and a small reduction in δ^13^C values, followed by a rise of 1.0‰ in δ^15^N before the completion of M2 development. This could indicate a considerable reduction in meat consumption, followed by a phase of physiological stress, as the increase in δ^15^N values is not paralleled by δ^13^C values. Indeed, skeletal evidence of mild cribra orbitalia, healed endocranial lesions and enamel hypoplasia confirm this individual’s exposure to physiological stress earlier in life, and potentially on an intermittent basis [55].

The greatest dietary instability across the life course is exhibited by Oxford Castle 4240 (female, 18–25 years) who presents the largest range of δ^15^N values (5.5‰) coupled with substantial variation in δ^13^C values (1.3‰). The most substantial perturbation occurred in the last increment (between 13 and 14 years old), with a drop of 4.0‰ to only 5.5‰ (lower than all of the faunal averages). Despite the magnitude of variation, consistent covariation with more marked fluctuations in δ^15^N values implicating changes in access to protein sources rather than physiological stress. However, this individual did present signs of childhood stress: both mild cribra orbitalia and hypoplastic defects [55].

Dietary variation in combination with periods of physiological stress may also be indicated among the incremental profiles. All Saints 58/2 (Male, 35+ years) showed a substantial range in δ^13^C (1.2‰) and δ^15^N (2.5‰), with co-variation up to the age of c. 5 years. For the rest of the curve, δ^13^C and δ^15^N values both decrease, with variation being more marked in the latter. This is difficult to interpret, but may relate to substantial meat consumption being replaced with other protein sources that have higher δ^13^C values, potentially crops grown in a landscape with higher baseline δ^13^C values, for example. δ^15^N values rise between the age of c. 10 and 13, coupled with a smaller reduction in δ^13^C. This could result from physiological stress at the point at which meat consumption was at its lowest, but it is followed by a smaller drop in δ^15^N values after the age of c. 13. This individual did experience physiological stress in childhood, evident from hypoplastic defects. They also have among the highest bone δ^15^N (12.5‰) and δ^13^C (-19.2‰) values of all 34 individuals studied here, and appear to have had a diet richer in animal protein in later life.

Oxford Castle 5787 (10–12 years, the only individual that did not reach adulthood) shows relatively substantial variation in δ^15^N (3.3‰) and δ^13^C (1.0‰) values but, in contrast to All Saints 58–2, access to meat must have been relatively limited, with a mean δ^15^N value of only 9.7‰ and a minimum of 7.8‰ indicating very little meat consumption in the last phase of life. There is no clear relationship between the proxies up to the age of c. 8, suggesting an unstable diet, but not only in terms of variable access to animal protein. In the last year of life, opposing covariance suggests a period of physiological stress. This individual presented lesions consistent with cribra orbitalia and scurvy at the time of death [55], suggesting limited access to both animal protein and fresh fruit and vegetables in the period leading up to their death.

Comparison of absolute δ^15^N and δ^13^C values between childhood (incremental data) and adulthood (bulk data) suggest all individuals either experienced long-term consistency in their sources of dietary protein (All Saints 53, 55, 63 Oxford Castle 5793, 5804) or consumed more/higher-trophic level protein sources in adulthood (All Saints 66, 58–2). The latter had different bone δ^15^N values suggesting overall differences in diet despite this similarity: All Saints 66 had the lowest of all 11th century samples (10.5‰) but All Saints 58–2 had a comparatively high bone δ^15^N value (12.5‰).

In all incremental profiles the influence of dietary change has been evaluated against variation arising from physiological stress. During childhood, this may arise from periods of ill health, starvation or particularly rapid growth [53, 54]. Oxford Castle 5793 (Female, 17–25 years) presented intermittent opposing covariance between c. 7–10 years, suggesting a period of physiological stress from which she later recovered. Individuals with the most varied profiles (All Saints 58–2, 63, Oxford Castle 4240, 5787) also presented skeletal evidence of physiological disturbance in childhood as all had linear enamel hypoplasia suggesting periods of growth disruption. All Saints 55 (female, 25+ years) had a notable dip in δ^15^N values between c. 10 and 14 years, potentially related to the nitrogen demands of adolescent growth, which can lower mean isotope value independent of diet [56]. It has also been possible to identify the potential impact of weaning in the earliest increments of All Saints 66 (a drop of 1.2‰ in δ^15^N and 0.4‰ in δ^13^C values in the first two increments, suggesting that weaning may have been extended in this individual, but had finished by the age of c. 4 years) and All Saints 63 (a sharp rise followed by gradual reduction in the nitrogen isotope curve that may indicate an increase in the reliance on breastfeeding around the age of c. 2 years). Extended weaning, which can ameliorate childhood starvation in times of food shortage [57], offers a possible explanation for these data.

Previous studies of incremental isotope profiles have tended to focus on substantial dietary shifts, for example the introduction of C4 crops as relief food during the Irish Famine [53], selective consumption of marine resources [58] and seasonal variation in subsistence practices [59], or have harnessed incremental data to document the weaning process [54, 60, 61]. In contrast, the data from Oxford present more modest variation, which is inherently more challenging to interpret unambiguously. Nonetheless, periods of opposing covariance attributable to physiological stress were few; rather, consistent covariation in isotope values suggests dietary variation was primarily responsible for many of the fluctuations observed. These brief periods of instability could, conceivably, relate to short term disruption to food supplies following the Conquest or to famines in AD 1005, 1016, 1044, 1070, 1082, 1087, 1097 and 1126 recorded in the Anglo-Saxon Chronicle [62], and the combined isotope and osteological data from Oxford Castle 5787 does indeed suggest death during a period of malnutrition, perhaps from starvation. It does not appear that other individuals experienced severe enough shortages to result in starvation, but the identification of extended weaning of young children in two cases suggests a response to food shortage was required at times. Some individuals showed a stable diet from earlier to later life (All Saints 53, 63 Oxford Castle 5793, 5804), while others showed more meat consumption in adulthood (All Saints 66, 55, 58–2). The individuals that showed the greatest dietary variation also appeared to have less access to meat overall through early life (Oxford Castle 4240 and 5787). These data confirm the presence of diets with a consistently substantial or increasing component of animal products among Oxford residents with evidence for dietary stress of a short-term nature. The data also caution against over-generalisation of diets during the Conquest by revealing that individual experience remained highly variable.

**Fig 8:**
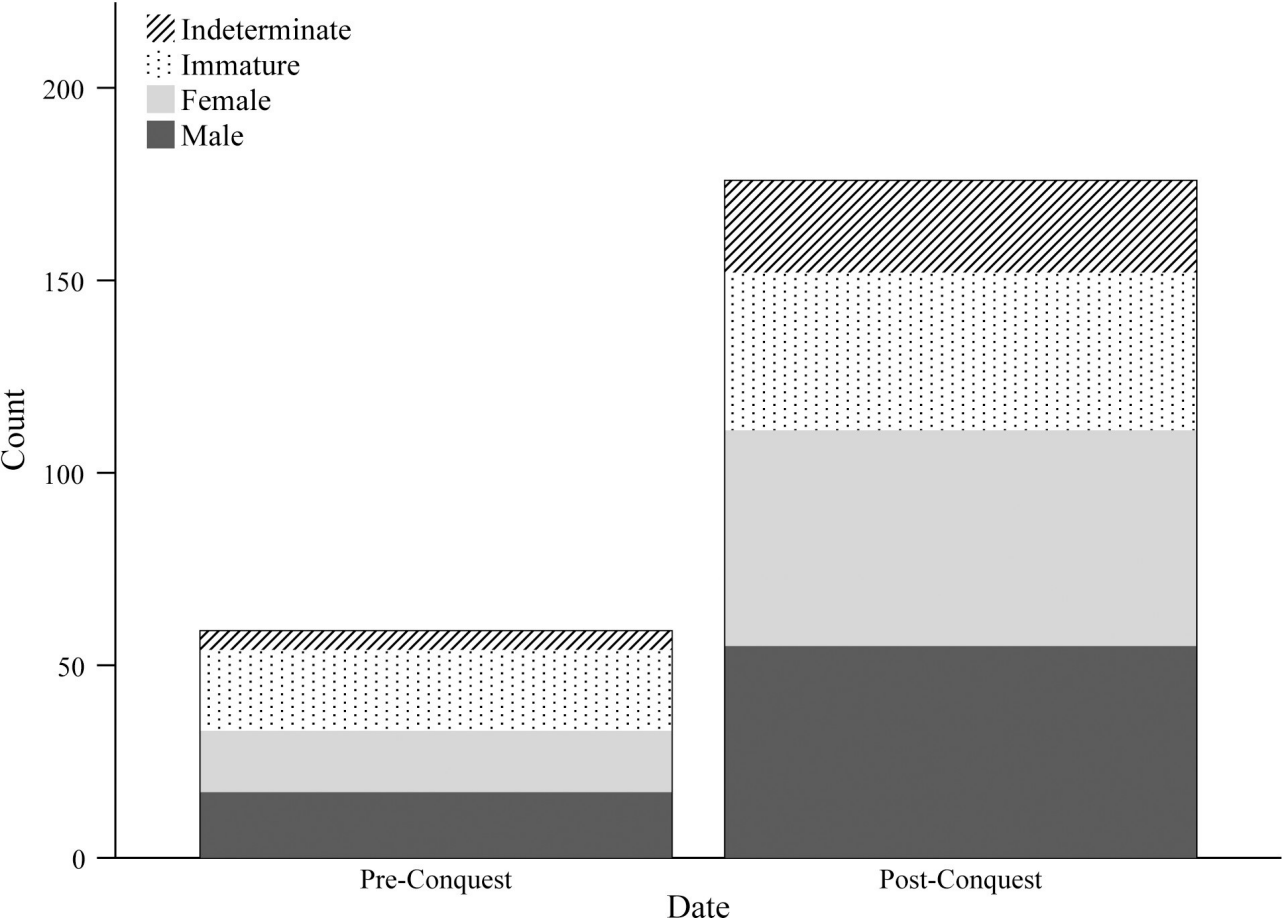
Sex profile of human remains included in the osteological assessment. Intermediate individuals are adults for whom sexually-dimorphic traits provided an inconclusive assessment. Immature individuals were not assigned a sex.

**Fig 9:**
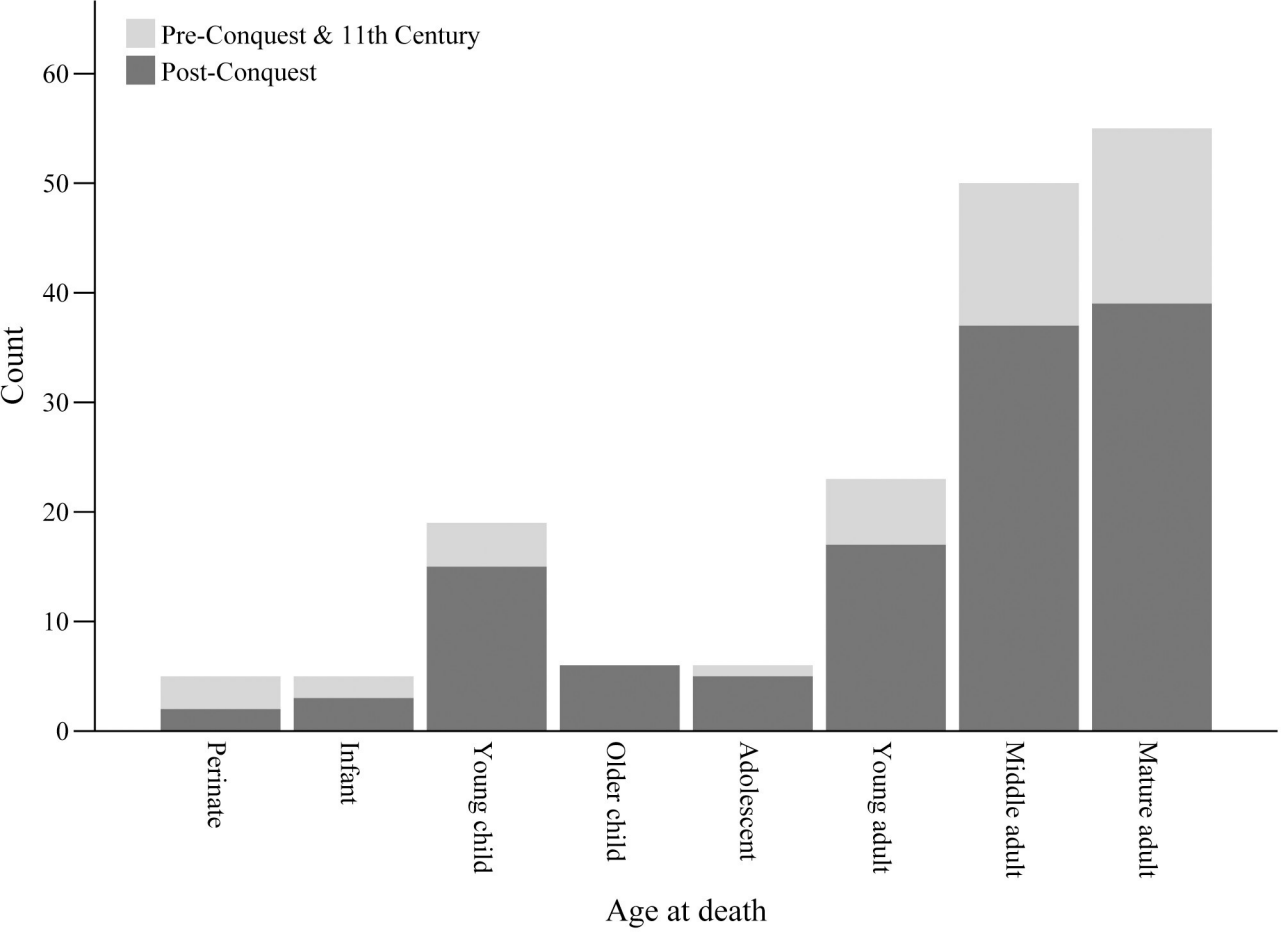
Age profile of human remains included in the osteological assessment. Age categories are as follows: perinate <1 month; infant 1 month-1 year; young child 1–5 years; older child 6–11 years; adolescent 12–17 years; young adult 18–30 years; middle adult 30–45 years; mature adult >45 years.

Analysis of human remains offers a final set of data to examine evidence for diet across the Norman Conquest. Osteological assessment of dental pathology and physiological stress-related conditions reflects aspects of diet at the population-level but also offers insight into individual experience, and is thus complementary to the data already explored in this paper. The demographic profile of the osteological sample included males, females and children of all ages (n = 235). The two chronological samples were evenly split between males and females; the combined pre-Conquest and 11^th^-century sample comprised 17 males and 16 females, and the post-Conquest sample 55 males and 56 females (Fig 8). The age-at-death profiles of the two cohorts were also largely consistent (Fig 9). These demographic similarities facilitate valid comparison of dental and physiological health between the two periods.

**Fig 10:**
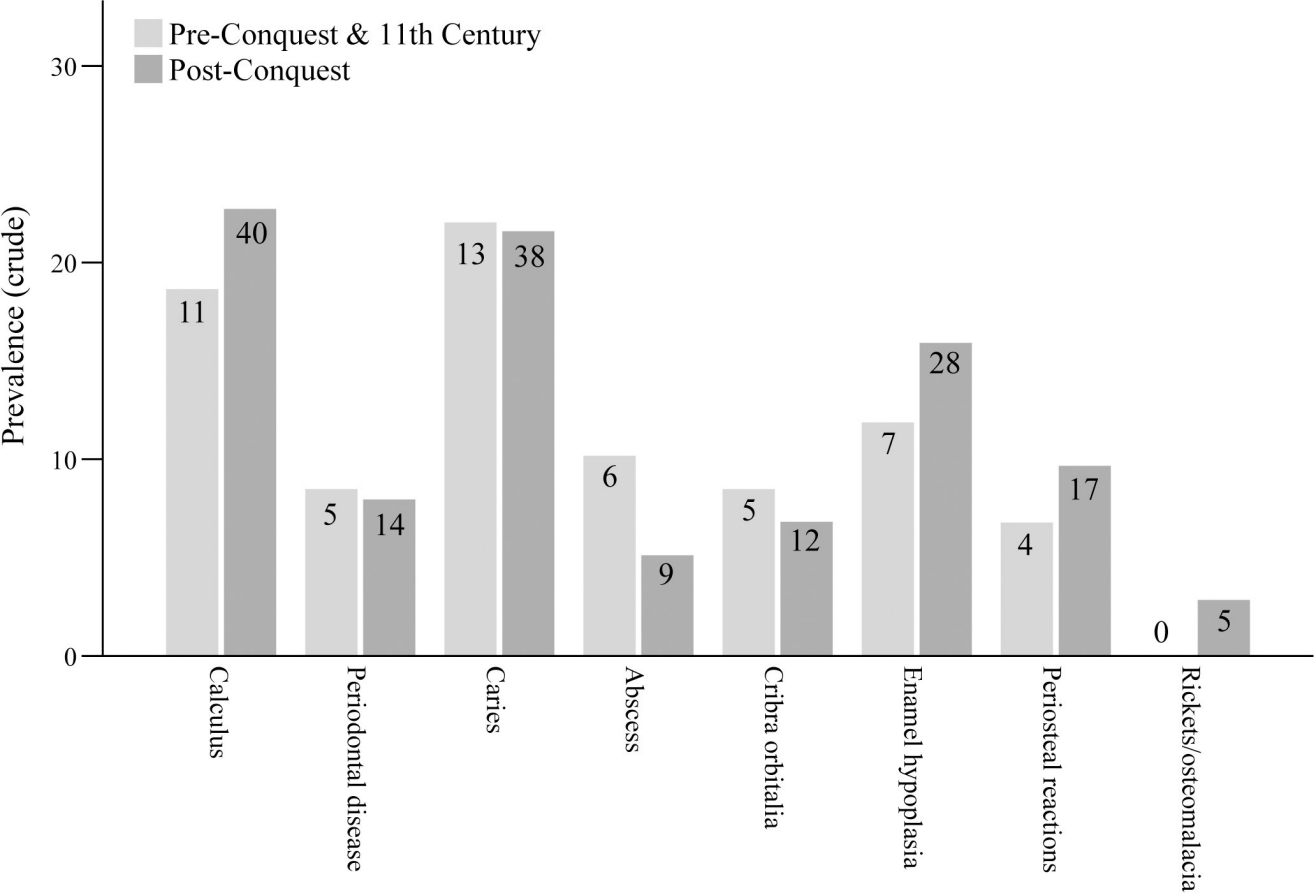
Crude prevalence of four dental pathologies and four markers of physiological stress in the Oxford human remains sample. n is indicated within each bar.

The prevalence of four dental pathologies and four markers of physiological stress are presented in Fig 10. Prevalence of calculus was slightly higher in post-Conquest individuals and rates of abscess lower, but neither attained statistical significance (Two-tailed Χ^2^ = 0.434, p = 0.510; Χ^2^ = 1.890, p = 0.169, respectively. α = 0.005. Analysis was undertaken in SPSS 25. S6 Table). Similar rates of periodontal disease and caries were observed in both samples. Overall, these findings suggest limited impact of the various dietary changes identified throughout this paper on the general dental health of the populace. This finding is pertinent in several specific ways. Calculus formation, for example, has been widely associated with diets high in protein, through an indirect increase in the alkalinity of the oral environment. A range of other, non-dietary, factors have also been implicated in calculus formation, however, including phosphate and calcium concentrations in saliva, fluid consumption and individual characteristics of the oral microbiome in addition to oral hygiene practices such as tooth brushing which retard the accumulation of dental plaque [63]. The similar rates of periodontal disease observed in the two cohorts is as expected, as there is a strong association between the two oral diseases [64]. Formation of dental caries is also a multifactorial process. The consumption of complex carbohydrates such as sugars is a major cause of caries formation, in combination with oral pH, dental morphology and environmental exposure to fluoride, among other factors [65]. The data examined here show similar patterns for both periods, providing no evidence of widespread changes in the consumption of carbohydrates. Thus, overall, these osteological findings are not consistent with a substantial overall change in the proportion of protein and/or carbohydrate consumed across the Conquest, but they do not preclude a change in the sources of these dietary constituents, for example a preference for pork over protein from herbivorous animals.

The prevalence of the four skeletal pathologies associated with physiological stress did not differ substantially between the pooled pre-Conquest and 11th-century sample and the post-Conquest sample. In all cases prevalences were generally low, in particular for linear enamel hypoplasia, which has been reported in between 20–40% of individuals from other medieval skeletal populations [66]. Rickets was rare, and only one case of possible scurvy was recorded, in Oxford Castle 5787 [55]. While the sample sizes here are small, the populations studied appear demographically representative and were not subject to levels of elevated age-specific mortality which would indicate hidden frailty (and therefore a potentially paradoxical lack of stress markers among a group experiencing extreme levels of physiological stress). As such these prevalences of skeletal stress markers do suggest that, overall, physiological stress levels were consistently low throughout the entire pre-, peri- and post-Conquest period in Oxford.

Historical evidence provides a mixed picture of life in 10th-12th century Oxford with which to contextualise these data concerning food supply and dietary change. Domesday Book indicates a substantial number of properties in the town were ‘waste’ (abandoned or dilapidated properties no longer providing a source of income to their owners), and therefore presumably unoccupied, implying a population decline of up to half from the immediate pre-Conquest period. However, evidence of increasing payments to the crown suggests rising prosperity due to economic development, and continuity of landholding within the city is suggestive of economic stability [67]. Archaeological evidence demonstrates the building up of street frontages in the late 11th century, suggesting an increasing range of economic activities were taking place, and perhaps, higher population density. On this basis, Munby [67] has suggested that, while Oxford had lost much of its political significance by the late 11th century (noting in particular that ‘wasted’ properties most commonly relate to the holdings of major landowners rather than those of townspeople), the town continued to grow and diversify economically. Thus, the various strands of our analysis, which suggest a greater uniformity of supply of foodstuffs to Oxford’s population following the Conquest, is consistent with suggestions that its negative impacts on everyday life were limited. While documents suggest land was laid to waste in Oxfordshire to thwart any Midland resistance, the Domesday survey suggests its impact was brief, so we would not expect there to have been a long-term disruption to food supply. Indeed, within the wider region, the remarkable lack of Domesday references to waste in Oxfordshire as a whole [68] implies that any disruption to rural life and economy in the town’s hinterland was also short-lived. Notably, the only proxy examined here with the potential to reveal short-term disruption in diet revealed substantial variation between individuals, suggesting some were indeed exposed to short-lived periods of food insecurity. A more developed urban economy with better, more regular access to resources for more people may well have been one outcome of the Conquest, but the data presented here suggest it did not result in a marked improvement or deterioration in the general health of the people of Oxford over the course of c. 300 years.

## Conclusions

The Norman Conquest of England is a well-known historical event, but its impact on the everyday lives of the English population is not well understood. This study has explored both long- and short-term change in diet across the period of the Norman Conquest in Oxford, demonstrating the potential of a multiproxy method comprising analysis of organic residues from pottery, bulk isotope analysis of faunal and human remains, incremental isotope analysis of human dentine, and palaeopathological assessment of human remains. The combination of these techniques allows different elements of diet, foodways and health to be understood at different temporal scales and levels of resolution. Diet provides an intimate link to individual experiences of these key points of transition and therefore the application of scientific techniques provides a unique source of evidence with which to revise traditional historical narratives.

Our findings, in combination, have revealed a pattern of increasing intensification and marketisation across various areas of economic practice with a much lesser and more short-term impact of the Conquest on everyday lifestyles that is suggested by documentary sources. Evidence of preferences for certain foodstuffs and cooking techniques suggests that fashions documented among elite classes and at specific rural ‘provider’ sites were also adopted into the everyday lives of those living in towns. Yet the impact of restricted access to food, particularly during the short-term famines documented across the 11th and 12th centuries, indicates markedly varied individual experience underlying the broader homogenising trends seen in the long-term data. This study deepens understanding of the socio-economic contexts of transition by elucidating the undocumented, everyday implications of the large-scale political and cultural transformations accompanying the Norman Conquest of England.

## Supporting information

S1 Data (DOCX)

S1 Table Summary of the residue analysis of the early medieval pottery. (DOCX)

S2 Table Sample details and stable isotope results for animal bone collagen. (DOCX)

S3 Table Sample details and stable isotope value results for human remains. (DOCX)

S4 Table Summary data from the nine incrementally-sampled second molars from All Saints (AST) and Oxford Castle (OXC). a. Data from bone collagen failed to meet quality control criteria.
(DOCX)

S5 Table Full incremental δ^13^C and δ^15^N isotope data. (DOCX)

S6 Table Prevalence of calculus and abscesses by period. (DOCX)
