# Correction: The dietary impact of the Norman Conquest: A multiproxy archaeological investigation of Oxford, UK

**DOI:** 10.1371/journal.pone.0239640

**Published:** 2020-09-17

**Authors:** Elizabeth Craig-Atkins, Ben Jervis, Lucy Cramp, Simon Hammann, Alexandra J. Nederbragt, Elizabeth Nicholson, Allie Rae Taylor, Helen Whelton, Richard Madgwick

The following information is missing from the Funding statement: The NERC (Reference: CC010) and NEIF (www.isotopesuk.org) provided support in the form of funding and maintenance of the GC-QToF MS and GC-C-IRMS instruments used for this work.

The Acknowledgements section is missing from the paper. The authors would like to make the following Acknowledgements: The authors would like to thank John Cotter (Oxford Archaeology) and David Moon (Oxfordshire Museum) who kindly assisted with sample access and selection. Analytical work was conducted at the Stable Isotope Facility of Cardiff University’s School of Earth and Ocean Sciences, with sample preparation undertaken at the Cardiff University Bioarchaeology Laboratory (carbon and nitrogen isotope analysis). Organic residue analysis was undertaken at the Organic Geochemistry Unit laboratories at the University of Bristol. We are also grateful to Will Barrow and Rachel Mott for assistance with isotope sample preparation and preliminary data analysis, Louise Loe and Rebecca Nicholson of Oxford Archaeology for access to data from Oxford Castle prior to its publication in 2019, and to Julia Beaumont for discussions relating to the incremental dentine methodology. Sincere thanks are due to Aleks McClain, Naomi Sykes and the other members of the steering group of the AHRC-funded Archaeologies of the Norman Conquest Network for their support, and for providing an opportunity to discuss this research during its development. Comments on draft versions by Naomi Sykes, Paul Halstead and Caroline Jackson were gratefully received, as were comments from anonymous reviewers.
