# Supplementary material for: The dietary impact of the Norman Conquest: A multiproxy archaeological investigation of Oxford, UK
Source: PLoS One. 2020 Jul 6;15(7):e0235005. doi: 10.1371/journal.pone.0235005 (PMC7337355; doi:10.1371/journal.pone.0235005)
Supplement: S1 Data — (DOCX) [file pone.0235005.s001.docx]

**S1 Materials and Methods**

**Materials**

**Organic residue analysis of ceramics**

Forty-one samples of pottery were selected for organic residue analysis from four sites in Oxford. All are either currently, or will be, archived by Oxford Museums Service at their Research Centre, Standlake, Oxfordshire. These comprised 11 sherds of pre-Conquest Late Saxon Shelly Ware (OXB; dated c. AD 900-1050), 11 sherds of pre-Conquest St Neots Ware (OXR; dated c. AD 925-1050), 14 sherds of post-Conquest Oxford Ware (OXY; dated c. AD 1050-1250) and five sherds of post-Conquest Cotswold Ware (OXBF; dated c. AD 1050-1250). These are the principal ceramic types used in Oxford during the early medieval period and have the benefit of being closely dateable based on the scheme developed by Mellor [1]. Sherds were selected from jars (cooking pots) which showed evidence of use in cooking. The majority of sherds were from the body of the vessel, a smaller number from the base and the rim. Where possible, [2]. Where possible, sherds were selected from contexts which could be closely dated on stratigraphic grounds. The organic residue data offer direct evidence of foodstuffs stored, prepared and served in the vessels examined.

**Faunal remains**

The faunal assemblage comprised 60 animals with equal numbers of cattle (n=20), pigs (n=20) and caprines (n=20) (Table S1.1). All specimens were sampled for isotope analysis of δ^13^C and δ^15^N *in situ* at the Oxfordshire Museum Resource Centre, Standlake, Oxfordshire. Wherever possible, repeat sampling of sided zones of specific elements from each site (e.g. medial surface of distal diaphysis of pig tibiae) was undertaken in order to ensure that each sample represented a distinct individual. Compromises had to be made in the sampling strategy, as faunal assemblages were not large enough to repeat sample the same sided zones for all sites. In some instances, mandibles were sampled so that dental attrition could be used to ensure that specimens derived from different individuals. In other instances, it was necessary to select two (or occasionally more) sided elements from a single taxon to provide sufficient samples. However, the lack of overlapping results indicates that this is not likely to have resulted in multiple samples from the same animal. All animals were sub-adult or older and therefore nursing signals should not impact isotope values. None of the samples were directly dated, therefore the chronological assignment was based on stratigraphic phasing presented in site reports. In consequence, it was not possible to identify a group of animals that straddled the period of the Conquest. These data provided direct evidence of livestock management strategies and a baseline from which to reconstruct human diets.

**Table S1.1. The animal bone sampled for isotope analysis.**

| **Site** | **Pre-Conquest** | | | **Post-Conquest** | | | ***Total*** |
| --- | --- | --- | --- | --- | --- | --- | --- |
|  | Cattle | Pig | Sheep/ Goat | Cattle | Pig | Sheep/ Goat |  |
| St Aldate’s Police Station^a^ | 0 | 0 | 0 | 10 | 10 | 10 | *30* |
| All Saints | 5 | 5 | 5 | 0 | 0 | 0 | *15* |
| Oxford Castle | 5 | 5 | 5 | 0 | 0 | 0 | *15* |
| ***Total*** | *10* | *10* | *10* | *10* | *10* | *10* | *60* |

1. Includes the Police Station and Land Adjacent to the Police Station sites.

**Human remains**

A sample of human remains was collected for stable isotope analysis and osteological assessment of both dental pathology and non-specific markers of physiological stress (Table S1.2). The full assemblage comprised 284 individuals from eight excavations, but accessibility of the original skeletal material, quality of published and unpublished data and financial resources available for isotope analysis necessitated the selection of sub-samples for each part of the analysis. Radiocarbon dates were available for 19 individuals from five sites (Table S1.3) and, alongside detailed stratigraphic data, enabled the sample to be allocated to pre-Conquest, 11th-century and post-Conquest groups.

**Table S1.2. The total sample of human remains from which sub-samples were selected for osteological assessment and stable isotope analysis.**

| **Site** | **Pre-Conquest** | **11th Century** | **Post-Conquest** | ***Total*** |
| --- | --- | --- | --- | --- |
| St Aldate’s Church | 10 |  |  | *10* |
| All Saints’ Church |  | 19 | 61 | *80* |
| Bonn Square |  |  | 111 | *111* |
| Christ Church Cathedral | 36 |  |  | *36* |
| Christ Church Cloister | 17 |  |  | *17* |
| Christ Church Tom Quad |  |  | 4 | *4* |
| Oxford Castle | 2 | 11 |  | *13* |
| Westgate (barbican ditch) |  |  | 13 | *13* |
| **Total included in osteological analysis** | 29 | 30 | 176 | 235 |
| **Total included in bulk isotope analysis** | 7 | 15 | 16 | 38 |
| **Total included in incremental isotope analysis** |  | 8 | 1 | 9 |
| ***Grand Total*** | ***65*** | ***30*** | ***189*** | ***284*** |

**Table S1.3. Radiocarbon dates for human remains included in this study [3-5].**

| **Site** | **Radiocarbon dates** | | |
| --- | --- | --- | --- |
|  | **Skeleton number** | **Date BP** | **Calibrated date (95% confidence)** |
| All Saints’ Church | 55 | 920+/-70 | 980-1270 |
|  | 57 | 870+/160 | 1010-1290 |
|  | 67 | 650+/-70 | 1250-1430 |
| Christ Church Cathedral | 402 | 1369+/-32 | 620-690 |
|  | 418 | 1197+/-33 | 690-960 |
|  | 277 | 1135+/-29 | 780-990 |
| Christ Church Cloister | 96 | 1160+/-40 | 775-976 |
|  | 119 | 1150+/-40 | 779-982 |
|  | 123 | 1110+/-40 | 827-1009 |
|  | 127 | 1250+/-40 | 668-884 |
| Oxford Castle | 4238a | 1046+/-30 | 874-934 |
|  | 4238b | 952+/-15 | 983-1013 |
|  | 4249 | 1006+/-30 | 914-974 |
|  | 4200 | 939+/-20 | 991-1031 |
|  | 5779 | 929+/-15 | 1006-1036 |
|  | 5794 | 967+/-35 | 948-1019 |
| St Aldate’s | 846 | 1147+/-28 | 780-990 |
|  | 835 | 1210+/-36 | 690-940 |
|  | 855 | 1107+/-28 | 880-1000 |

**Human osteological analysis**

New osteological analysis was conducted on human remains from All Saints’ Church at the Oxfordshire Museum Resource Centre, Standlake, Oxfordshire, and collated with published data from St Peter-le-Bailey, Bonn Square; Christ Church (Cloister and Tom Quad); St Aldate’s Church; and Oxford Castle. Remains from Christ Church Cathedral and the stratigraphically-higher burials from St Aldate’s were omitted from osteological analysis as the original assessment had been undertaken *in situ* without systematic recording of skeletal or dental pathology, and the majority of material was never lifted and thus was inaccessible for reanalysis [6]. Westgate was omitted as the material retained post-excavation comprised only femora. This resulted in a total sample of 235 individuals for osteological assessment. Of these, a smaller proportion could be confidently dated to before the Conquest (n=29) and to the 11th century (n=30) than to the post-Conquest period (n=176). The pre-Conquest and 11th-century groups were therefore combined for analysis. These data provide an insight into skeletal health and dietary pathologies affecting the dentition.

**Bulk isotope analysis of bone collagen**

A sub-sample of 38 adults from the human remains were selected for stable carbon and nitrogen isotope analysis of bone collagen to reconstruct individual diets, from which two 11th-century individuals from Oxford Castle had to be omitted due to poor C:N ratios (Table S1.4). Material was sampled *in situ* at the Oxfordshire Museum Resource Centre, Standlake, Oxfordshire. Ribs were targeted for analysis to minimise destruction of the material, but suitable samples could only be acquired from 13 individuals. For 18 further individuals, femora were sampled as this element has been shown to have a very similar turnover rate to ribs and therefore provides a comparable temporal signal [7]. The remaining samples came from two tibiae and a humerus, which have broadly comparable turnover rates, and a clavicle, which provides a longer-term averaged signal [7]. Similar consideration of turnover rates was not necessary for the animal bone assemblage as a shorter lifespan minimises this issue. The human bone collagen data provide direct evidence of diet averaged over several years prior to death.

**Table S1.4. The sub-sample of human remains sampled for bulk isotope analysis of bone.**

| **Site** | **Pre-Conquest** | **11th Century** | **Post-Conquest** | ***Total*** |
| --- | --- | --- | --- | --- |
| All Saints’ Church | 0 | 9 | 3 | *12* |
| Christ Church Cathedral | 6 | 0 | 0 | *6* |
| Oxford Castle | 1 | 2 | 0 | *3^a^* |
| Westgate | 0 | 0 | 13 | *13* |
| ***Total*** | *7* | *13^a^* | *16* | *36* |

- 1. Including two samples later omitted due to poor C:N ratios.

**Incremental isotope analysis of tooth dentine**

Nine individuals from All Saints (n=5) and Oxford Castle (n=4) were selected for incremental δ^13^C/δ^15^N isotope analysis of dentine to track patterns of early life diet and physiological stress. Eight individuals were dated to the 11th century and one (from All Saints) to the early post-Conquest period. All had also been sampled for bulk bone collagen analysis, facilitating comparison of data relating to early and later life. These data provide high-resolution insights into dietary change during childhood at an individual level across the period of the Conquest.

**Methods**

The following section provides a summary of each method, a justification of its potential and details of its application in the present study.

**Organic residue analysis of ceramics**

Organic residue analysis of archaeological ceramics enables the extraction and identification of preserved organic compounds absorbed in the fabric of pottery vessels. This technique has proved particularly informative in assessing changing subsistence strategies and foodways in the past by permitting chemical identification of commodities that were processed, stored and cooked in ceramic cooking vessels through surviving traces of fats, waxes, oils and resins [8, 9]. Differentiation of non-ruminant adipose (e.g. pork, chicken), ruminant adipose (e.g. beef, lamb, goat), ruminant dairy products and aquatic produce is possible, and plant oils and waxes can also be distinguished with varying degrees of specificity [9]. Chemical changes arising from the sustained heating of foodstuffs can also be identified [10]. Most recently, techniques have been developed for the detection of major cereal crops, although optimal survival of these biomarkers would be expected from pottery buried in anoxic conditions [11].

Samples of approximately 2g were selected from 41 potsherds. The exterior surface was cleaned using a modelling drill to ensure that any modern contaminants from soil burial and excavation/post-excavation handling were removed. Cleaned potsherds were crushed and ground to a fine powder using pestle and mortar. Preserved organic molecules were extracted using a mixture of organic solvents (methanol and chloroform). Obtained total lipid extracts (TLEs) were then screened using gas chromatography (GC) in order to determine the lipid concentrations. The samples with preserved organic compounds were further analysed using GC-mass spectrometry (GC-MS) and GC-combustion-isotope ratio mass spectrometry (GC-C-IRMS) in order to identify the presence and origins of potentially-preserved organic molecules. A significant lipid concentration (>5 μg g^-1^ of potsherd) was detected in 63.4% (26/41) of the sampled potsherds. This lipid concentration limit represents the minimum which can be reliably attributed to remnants of ancient food processing rather than modern contamination.

A combination of good preservation based on both hood recovery rate and high lipid content and the relatively young age of our samples suggested that cereal biomarkers could be preserved despite the absence of anoxic conditions. Therefore, a targeted analysis was also undertaken to identify specific cereal biomarkers. Aliquots of TLEs produced by the previous method were purified chromatographically using an NH_2_-SPE method to remove the major and potentially-interfering lipids, i.e. free fatty acids and triacylglycerols and allow an enrichment of alkylresorcinols and sterols in one fraction. An alkylresorcinol standard was added to the lipid extracts prior to purification to validate performance of the method. The purified lipids were derivatised by trimethylsilylation, screened by GC and subsequently analysed by GC coupled to high resolution mass spectrometry (GC/Q-TOF MS) to identify alkylresorcinols, sterols, stanols, long-chain alcohols, monoacylglycerols and diacylglycerols.

**Stable isotope analysis**

Isotope analysis of δ^13^C and δ^15^N from bone collagen and tooth dentine is a well-established means of analysing the proportional contribution of plant-, animal- and marine-derived protein to diet, thus providing a vital source of direct evidence for consumption patterns among past human and animal populations [12]. δ^13^C isotope values in bone collagen are principally determined by the sources of plant foods and the consumption of marine resources. δ^15^N isotope values primarily reflect trophic level, thereby revealing sources and proportional quantity of dietary protein [13] but can also reflect health status [14] and farming practices, such as manuring [15], exploitation of salt marsh [16, 17] or the proportion of animal protein fed to omnivores [18]. To facilitate exploration of long- and short-term variation in diets among both individuals and the population of Oxford, this study employed bulk sampling of human and animal bone collagen alongside incremental sampling of human tooth dentine. Bone has a variable, but generally slow turnover rate [7], therefore provides a dietary signal that has been averaged over a period of years prior to death. By contrast, dentine provides a short-term signal for early life diet, as it mineralises a matter of days after secretion during ontogeny and undergoes very little remodelling thereafter. Analysis of increments of dentine generates multiple isotope values for each individual which represent a rolling short-term average of dietary change during the period of the tooth’s development and mineralisation [19]. The enhanced temporal resolution of these dental isotope signals means that changes in health status, particularly in terms of physiological stress, can also be observed through incremental analysis.

**Sample preparation**

For isotope analysis of animal and human bone collagen, fragments of bone weighing between 0.5 and 1.0g were sampled from each specimen using an Argofile precision drill with diamond wheel attachment. The outer surface of each bone sample was abraded using a diamond burr to remove any adhering contaminants. Samples then proceeded to the collagen extraction process (see below). For incremental analysis of dentine collagen, a single mandibular second molar was selected, avoiding teeth with heavy wear or evidence of dental pathology. Each molar was extracted from the mandible and tooth roots were abraded with a diamond burr to remove adhering contaminants. All enamel was then mechanically removed from the crown using a burr. For individuals over the age of 25, pulp chambers were exposed and reamed using a conical burr to remove any secondary dentine prior to demineralisation, thus ensuring that isotope values relate to childhood rather than later remodelling [20]. Once demineralisation of the entire tooth had been completed, sequential transverse sections of dentine were sampled from the occlusal surface to the root apex (see below) resulting in between 10 and 21 increments per individual. The sampling of a second molar provides the potential to track dietary changes between the approximate ages of 2.5 and 15.5 years [21] depending on dental attrition and individual variation in development, which is expected to be minimal. In eight of the nine samples second molars were fully mineralised and had erupted to full occlusion with root apices closed. One sample (OXC102) was from an adolescent individual was still developing at time of death and therefore provided signals up to the age of approximately 11.5 years.

**Collagen extraction and mass spectrometry**

The collagen-extraction protocol followed a modified version of the Longin method [22]. Each sample was placed in a pyrex test tube and demineralised in 8ml of 0.5M HCl at 4°C. The HCl was changed approximately every 2 days until demineralisation was complete. Demineralised specimens were thoroughly rinsed in deionised water. Demineralised teeth were sliced into transverse increments of approximately 1-2 mm breadth using a scalpel. Each increment was then placed in a separate pyrex test tube. Each incremental sample and all bone samples were then gelatinized in a pH3 solution of HCl at 70°C for 48 hours. The supernate containing the soluble collagen was collected using an ezee-filter and transferred to a polypropylene test tube before freeze-drying. Once freeze dried, 0.75mg of collagen was weighed into tin capsules and analysed in duplicate or triplicate where necessary. Eleven of the 137 incremental samples did not yield sufficient collagen for duplicate analysis and a further six produced too little collagen for any analysis (see S6 table). All 96 bone samples produced sufficient collagen for replicate analysis, but two samples from Oxford Castle were excluded due to poor C:N ratios. Mass spectrometry was undertaken using a Flash 1105 elemental analyser coupled to a ThermoFinigan Delta V Advantage was used in analysis. Laboratory standards comprised caffeine and supermarket gelatine with a mean 1𝜎 reproducibility of 0.080 for δ^15^N and 0.092 for δ^13^C. Carbon and nitrogen and isotope ratios (δ^13^C, δ^15^N) are reported in per mil (‰) relative to VPDB and AIR standards respectively. Two bone samples from Oxford Castle were excluded due to a poor C:N ratio.

**Osteoarchaeological assessment**

The quality and composition of an individual’s diet is a key factor in growth and development, disease resistance and reproductive function [23, 24]. Malnutrition – the deficiency of certain components in the diet – can lead to metabolic diseases which can be detected in skeletal remains [25]. Moreover, the direct interaction between food and dentition in the mouth also means that diet can influence the occurrence and progression of dental pathology [26]. While basic access to sufficient nutrition is an important factor in the prevalence of dietary-related disease in the past, a suite of socio-cultural factors, including differential access to certain foodstuffs based on age, gender or status, religious proscriptions and fashions in cuisine may also be influential [26, 27]. A range of pathological changes in the skeleton were considered in this study to examine the impact of dietary change across the Conquest on the prevalence of skeletal and dental pathology: the metabolic disorders scurvy and rickets; and the non-specific skeletal stress markers linear enamel hypoplasia, cribra orbitalia and periosteal reactions of the major long bones. Dental calculus, caries, periodontal disease and ante-mortem tooth loss were also assessed as diet is strongly implicated in their formation [28], but they have also been linked to a range of specific disease processes, which might reveal systemic health [29]. As some of the data utilised here were obtained from archive records of collections which had been reburied, it was impossible to undertake new data collection. In consequence, the range of pathological conditions we were able to consider was pre-determined and the methods of analysing the data were restricted to crude prevalence (number of cases of pathology as a proportion of total population). Presence and absence were examined as severity of the conditions was recorded inconsistently.

New osteological data were obtained from reanalysis of skeletons from All Saints. For all other sites, data were obtained from a combination of published and archived records. Methods for primary data collection were selected for consistency across the dataset. All assessment was undertaken macroscopically. Sex assessment was based on dimorphic traits of the pelvis and skull [30, 31]. Age assessment in adults was obtained from degeneration of the pubic symphysis [32] and auricular surface [33], and in immatures from dental development [22, 34] and epiphyseal fusion [35]. Pathological changes to the skeleton were recorded based on criteria set out in Brickley and McKinley [30] Roberts and Manchester [36] and Ortner and Putschar [37] and dental pathology was identified in line with Brothwell [38], Powell [39], Ogden [40] and Reid and Dean [41]. Although initially recorded by severity and location [42], pathological data were converted to presence/absence for inclusion in the present study.

**References**

1. Mellor M. A synthesis of middle and late Saxon, medieval and early post-medieval pottery in the Oxford region. Oxoniensia. 1994; 59: 17-217.
2. Skibo J. Understanding pottery function. New York: Springer; 2013.
3. Dodd A. Oxford before the University: The late Saxon and Norman archaeology of the Thames crossing, the defences and the town*.* Thames Valley Landscapes Monograph No. 17. Oxford: Oxford Archaeology; 2003.
4. Pollard AM, Ditchfield P, Piva E, Wallis S, Falys C, Ford S. ‘Spouting like cockle amongst the wheat’: The St Brice’s Day Massacre and the isotopic analysis of human bones from St John’s College, Oxford. Oxford J of Archaeol. 2012;31 (1): 83-102. doi: 10.1111/j.1468-0092.2011.00380.x.
5. Pollard AM, Ditchfield P. Stable isotope analysis of the human bones from Oxford Castle. In: Munby J, Norton A, Poore D, Dodd A. *Excavations at Oxford Castle 1999-2009*, editors. Thames Valley Landscapes Monograph 44. Oxford: Oxford Archaeology; 2019.
6. Durham B. Archaeological investigations in St Aldate’s, Oxford. Oxoniensia. 1977; 42: 83-203.
7. Fahy G, Deter C, Pitfield J, Miszkiewicz J, Mahoney P. Bone deep: Variation in stable isotope ratios and histomorphometric measurements of bone remodelling within adult humans. J Archaeol Sci. 2017; 87: 10-16. doi: 10.1016/j.jas.2017.09.009.
8. Evershed RP. Organic residue analysis in archaeology: the archaeological biomarker revolution. *Archaeometry*. 2008;50: 895-924. doi:10.1111/j.1475-4754.2008.00446.x.
9. Roffet-Salque M, Dunne J, Altoft D, Casanova E, Cramp L, Smyth J, Whelton H, Evershed R. From the inside out: Upscaling organic residue analyses of archaeological ceramics. J Archaeol Sci Rep. 2017; 16: 627-64. doi: 10.1016/j.jasrep.2016.04.005.
10. Kolattukudy PE, Croteau R, Buckner JS. Biochemistry of plant waxes. In: Kolattukudy PE, editor. Chemistry and biochemistry of natural waxes. New York: Elsevier; 1976. pp. 289-347.
11. Hammann S. Cramp L. Towards the detection of dietary cereal processing through absorbed lipid biomarkers in archaeological pottery. J Archaeol Sci. 2018;93: 74-81. doi: 10.1016/j.jas.2018.02.017.
12. Schoeninger MJ, DeNiro M, Tauber H. Stable nitrogen isotope ratios of bone collagen reflect marine and terrestrial components of prehistoric human diet. Science. 1983; 220: 1381–1383. doi: 10.1126/science.6344217.
13. DeNiro MJ, Epstein S. Influence of diet on the distribution of carbon isotopes in animals. Geochim Cosmochim Ac. 1978; 42: 495–506. doi: 10.1016/0016-7037(78)90199-0.
14. Fuller BT, Fuller JL, Sage NE, Harris DA, O'Connell TC, Hedges REM. Nitrogen balance and δ15N: why you're not what you eat during nutritional stress. Rapid Commun Mass Sp. 2005; 19 (18): 2497–2506. doi: 10.1002/rcm.2090.
15. Stevens RE, Lightfoot E, Hamilton J, Cunliffe BW, Hedges, REM. One for the master and one for the dame: stable isotope investigations of Iron Age animal husbandry in the Danebury environs. Archaeol Anthrop Sci. 2013;5 (2): 95-109. doi: 10.1007/s12520-012-0114-3.
16. Britton K, Muldner G, Bell M. Stable isotope evidence for salt-marsh grazing in the Bronze Age Severn Estuary, UK: implications for palaeodietary analysis at coastal sites. J Archaeol Sci. 2008;35: 2111-2118. doi: 10.1016/j.jas.2008.01.012.
17. Madgwick R, Sykes N, Miller H, Symmons R, Morris J, Lamb A. Fallow deer (Dama dama dama) management in Roman South-East Britain. Archaeol Anthrop Sci. 2013;5 (1): 111-122. doi: 10.1007/s12520-013-0120-012Z.00000000011.
18. Madgwick R, Mulville J, Stevens RE. Diversity in foddering strategy and herd management in late Bronze Age Britain: an isotopic investigation of pigs and other fauna from two midden sites. Environ Archaeol. 2012*;*17 (2): 126-140. doi: 10.1179/1461410.
19. Beaumont J, Montgomery J. Oral histories: A simple method of assigning chronological age to isotopic values from human dentine collagen. Ann Hum Biol. 2015;42: 407–414. doi: 10.3109/03014460.2015.1045027.
20. Beaumont J, Montgomery J. Oral histories: A simple method of assigning chronological age to isotopic values from human dentine collagen. Ann Hum Biol. 2015;42: 407–414. doi: 10.3109/03014460.2015.1045027.
21. Al Qahtani SJ, Hector MP, Liversidge HM. Brief communication: The London atlas of human tooth development and eruption. Am J Phys Anthropol. 2010;142: 481–490. doi: 10.1002/ajpa.21258.
22. Brown T, Nelson DE, Vogel JS, Southon JR. Improved collagen extraction by modified Longin method. *Radiocarbon.* 1988;30: 171–177. doi: 10.1017/S0033822200044118.
23. Larsen CS. Bioarchaeology: Interpreting Behavior from the Human Skeleton. Cambridge: Cambridge University Press; 2015.
24. Moffat T,Prowse T. Human diet and nutrition in biocultural perspective: Past meets present. New York, Berghahn Books; 2014.
25. Brickley M, Ives R. The bioarchaeology of metabolic bone disease. Amsterdam: Elsevier; 2008. doi: 10.1016/B978-0-12-370486-3.X0001-7.
26. Hastorf CA. The social archaeology of food: Thinking about eating from prehistory to the present. Cambridge: Cambridge University Press; 2017.
27. Hillson S. Teeth*.* 2nd ed. Cambridge: Cambridge University Press; 2013.
28. Lieverse AR. Diet and the aetiology of dental calculus. Int J Osteoarchaeol. 1999;9: 219-232. doi: 10.1002/(SICI)1099-1212(199907/08)9:4<219::AID-OA475>3.0.CO;2-V
29. De Witte SN, Bekvalac J. Oral health and frailty in the medieval English cemetery of St Mary Graces. Am J Phys Anthropol. 2010;142 (3): 341-54. doi: 10.1002/ajpa.21228.
30. Brickley M, McKinley JI. Guidelines to the standards for recording human remains. IFA paper 7. Reading: BABAO and *IFA*; 2004.
31. White TD, Folkens PA. The human bone manual. New York: Elsevier; 2015.
32. Brooks S, Suchey JM. Skeletal age determination based on the os pubis: a comparison of the Acsádi-Nemeskéri and Suchey-Brooks methods. Human Evolut. 1990;*5* (3): 227-238. doi: 10.1007/BF02437238.
33. Lovejoy CO, Meindl RS, Pryzbeck TR, Mensforth RP. Chronological metamorphosis of the auricular surface of the ilium: a new method for the determination of adult skeletal age at death. Am J Phys Anthropol. 1985;68 (1): 15-28. doi: 10.1002/ajpa.1330680103.
34. Ubelaker DH. The estimation of age at death from immature human bone. In: Işcan MY, editor. Age markers in the human skeleton. Springfield, IL: Charles C Thomas; 1989. pp.55-69.
35. Scheuer L, Black S. The juvenile skeleton. New York: Elsevier; 2004.
36. Roberts C, Manchester K. The archaeology of disease. 2nd edition. Gloucester: Sutton Publishing; 1995.
37. Ortner DJ. Identification of pathological conditions in human skeletal remains. New York: Elsevier. 2003.
38. Brothwell DR. Digging up bones. London: British Museum (Natural History); 1963.
39. Powell ML. Analysis of dental wear and caries for dietary reconstruction. In Gilbert RI Jr, Mielke JH, editors. The analysis of prehistoric diet. Orlando, FL: Academic Press; 1985. Pp. 307-338.
40. Ogden A. Advances in the palaeopathology of teeth and jaws. In Pinhasi R, Mays S, editors. Advances in human palaeopathology. Chichester; Wiley and Sons; 2008. pp.283-307.
41. Reid DJ, Dean MC. Brief communication: the timing of linear hypoplasias on human anterior teeth. Am J Phys Anth. 2000;113 (1): 135-139. doi: 10.1002/1096-8644(200009)113:1<135::AID-AJPA13>3.0.CO;2-A.
42. Taylor A. Health and Diet Across the Norman Conquest: A Skeletal and Data Analysis of Anglo-Norman Burials from Oxfordshire, England. M.Sc Thesis, University of Sheffield. 2016.
