# Supplementary material for: The dietary impact of the Norman Conquest: A multiproxy archaeological investigation of Oxford, UK
Source: PLoS One. 2020 Jul 6;15(7):e0235005. doi: 10.1371/journal.pone.0235005 (PMC7337355; doi:10.1371/journal.pone.0235005)
Supplement: S1 Table — (DOCX) [file pone.0235005.s002.docx]

**S2 Table. Summary of the residue analysis of the early medieval pottery**.

| **Sample** | **Fabric** | **Lipid Concentration (µg g^-1^)** | **δ^13^C_16:0_ (‰)** | **δ^13^C_18:0_ (‰)** | **Δ^13^C (‰)** | **Attribution of animal fats** | **Additional information** | **Chronology** |
| --- | --- | --- | --- | --- | --- | --- | --- | --- |
| SN-1 | Oxford ware | 513 | -28.2 | -29.8 | -1.6 | Ruminant adipose | Plant biomarkers (sterols, stanols) | Post-Conquest |
| SN-2 | Oxford ware | 348 | -27.7 | -29.3 | -1.6 | Ruminant adipose | - | Post-Conquest |
| SN-3 | St Neots | 266 | -27.0 | -29.0 | -1.9 | Ruminant adipose | Plant biomarkers (sterols, stanols) | Pre-Conquest |
| SN-4 | St Neots | 16 | -27.4 | -30.4 | -2.9 | Mixed ruminant adipose/ ruminant dairy | - | Pre-Conquest |
| SN-5 | St Neots | 360 | -27.4 | -29.9 | -2.5 | Ruminant adipose | Plant biomarkers (sterols, stanols, *n-*alcohols), brassica epicuticular wax | Pre-Conquest |
| SN-6 | St Neots | 90 | -26.8 | -27.3 | -0.5 | Mixed non-ruminant adipose/ ruminant adipose | Plant biomarkers (sterols, stanols), brassica epicuticular wax | Pre-Conquest |
| SN-7 | Oxford ware | 649 | -25.5 | -25.1 | 0.4 | Non-ruminant adipose | - | Post-Conquest |
| SN-8 | St Neots | 182 | -26.8 | -28.7 | -1.9 | Ruminant adipose | Plant biomarkers (sterols, stanols) | Pre-Conquest |
| *SN-9* | *Cotswold ware* | *-* | *-* | *-* | *-* | *-* | *-* | *Post-Conquest* |
| SN-10 | Cotswold ware | 186 | -28.0 | -29.9 | -1.9 | Ruminant adipose | Plant biomarkers (sterols, stanols), brassica epicuticular wax | Post-Conquest |
| *SN-11* | *Oxford ware* | *-* | *-* | *-* | *-* | *-* | *-* | *Post-Conquest* |
| *SN-12* | *Oxford ware* | *-* | *-* | *-* | *-* | *-* | *-* | *Post-Conquest* |
| *SN-13* | *St Neots* | *13* | *-* | *-* | *-* | *-* | *Plant biomarkers (sterols, stanols)* | *Pre-conquest* |
| *SN-14* | *St Neots* | *8* | *-* | *-* | *-* | *-* | *Plant biomarkers (sterols, stanols)* | *Pre-Conquest* |
| SN-15 | St Neots | 8 | *-* | *-* | *-* | *-* | *-* | Pre-Conquest |
| SN-16 | Oxford ware | 173 | -26.0 | -25.1 | 0.9 | Non-ruminant adipose | - | Post-Conquest |
| SN-17 | Oxford ware | 441 | -26.7 | -28.2 | -1.5 | Ruminant adipose | Plant biomarkers (sterols, stanols) | Post-Conquest |
| *SN-18* | *Cotswold ware* | *-* | *-* | *-* | *-* | *-* | *-* | *Post-Conquest* |
| SN-19 | Cotswold ware | 619 | -27.0 | -28.2 | -1.2 | Ruminant adipose | - | Post-Conquest |
| SN-20 | St Neots | 371 | -27.7 | -29.9 | -2.2 | Ruminant adipose | Plant biomarkers (*n-*alcohols) | Pre-Conquest |
| SN-21 | St Neots | 52 | *-* | *-* | *-* | *-* | *-* | Pre-Conquest |
| SN-22 | St Neots | 2125 | -28.8 | -30.2 | -1.4 | Ruminant adipose | Plant biomarkers (sterols, stanols, *n-*alcohols) | Pre-Conquest |
| SN-23 | Oxford ware | 143 | -27.6 | -29.1 | -1.4 | Ruminant adipose | Plant biomarkers (sterols, stanols, *n-*alcohols), brassica epicuticular wax | Post-Conquest |
| *SN-24* | *Oxford ware* | *-* | *-* | *-* | *-* | *-* | *Plant biomarkers (sterols, stanols)* | *Post-Conquest* |
| SN-25 | Oxford ware | 28 | - | - | - | - | Plant biomarkers (sterols, stanols, *n-*alcohols), evidence for heating (ketones) | Post-Conquest |
| *SN-26* | *Oxford ware* | *-* | *-* | *-* | *-* | *-* | *Plant biomarkers (sterols, stanols)* | *Post-Conquest* |
| SN-27 | Oxford ware | 160 | -29.1 | -30.5 | -1.4 | Ruminant adipose | Plant biomarkers (sterols, stanols, *n-*alcohols), brassica epicuticular wax | Post-Conquest |
| SN-28 | Oxford ware | 157 | -29.2 | -30.9 | -1.7 | Ruminant adipose | Plant biomarkers (sterols, stanols, *n-*alcohols), brassica epicuticular wax | Post-Conquest |
| SN-29 | Cotswold ware | 992 | -27.2 | -27.8 | -0.7 | Mixed non-ruminant adipose/ ruminant adipose | Plant biomarkers (sterols, stanols) | Post-Conquest |
| SN-30 | Oxford ware | 1071 | -28.8 | -30.2 | -1.4 | Ruminant adipose | Plant biomarkers (sterols, stanols, *n-*alcohols) | Post-Conquest |
| SN-31 | Late Saxon Shelly ware | 1768 | -27.3 | -29.1 | -1.8 | Ruminant adipose | - | Pre-Conquest |
| SN-32 | Late Saxon Shelly ware | 254 | -26.2 | -26.7 | -0.6 | Mixed non-ruminant adipose/ ruminant adipose | Plant biomarkers (sterols, stanols, *n-*alcohols), evidence for heating (ketones) | Pre-Conquest |
| SN-33 | Late Saxon Shelly ware | 5380 | -27.6 | -29.4 | -1.8 | Ruminant adipose | Plant biomarkers (sterols, stanols), evidence for heating (ketones) | Pre-Conquest |
| SN-34 | Late Saxon Shelly ware | 1487 | -27.8 | -29.6 | -1.8 | Ruminant adipose | Plant biomarkers (sterols, stanols, *n-*alcohols), evidence for heating (ketones) | Pre-Conquest |
| SN-35 | Late Saxon Shelly ware | 1149 | -27.7 | -29.7 | -1.9 | Ruminant adipose | Plant biomarkers (sterols, stanols, *n-*alcohols), evidence for heating (ketones) | Pre-Conquest |
| *SN-36* | *Late Saxon Shelly ware* | *-* | *-* | *-* | *-* | *-* | *Plant biomarkers (sterols, stanols)* | *Pre-Conquest* |
| SN-37 | Late Saxon Shelly ware | 874 | -27.1 | -28.7 | -1.5 | Ruminant adipose | Plant biomarkers (sterols, stanols, *n-*alcohols), evidence for heating (ketones) | Pre-Conquest |
| *SN-38* | *Late Saxon Shelly ware* | - | *-* | *-* | *-* | *-* | *-* | *Pre-Conquest* |
| SN-39 | Late Saxon Shelly ware | 549 | -27.3 | -29.9 | -2.6 | Ruminant adipose | Plant biomarkers (sterols, stanols, *n-*alcohols), beeswax | Pre-Conquest |
| SN-40 | Late Saxon Shelly ware | 25 | -27.6 | -33.4 | -5.8 | Ruminant dairy | Plant biomarkers (sterols, stanols) | Pre-Conquest |
| *SN-41* | *Late Saxon Shelly ware* | *-* | *-* | *-* | *-* | *-* | *Plant biomarkers (sterols, stanols)* | *Pre-Conquest* |

Samples which did not meet the required lipid concentration standards are italicised.
