# Supplementary material for: The dietary impact of the Norman Conquest: A multiproxy archaeological investigation of Oxford, UK
Source: PLoS One. 2020 Jul 6;15(7):e0235005. doi: 10.1371/journal.pone.0235005 (PMC7337355; doi:10.1371/journal.pone.0235005)
Supplement: S2 Table — (DOCX) [file pone.0235005.s003.docx]

**S3 Table. Sample details and stable isotope results for animal bone collagen.**

| **Sample** | **Site** | **Context** | **Date** | **Taxon** | **Element** | **Side** | **δ^13^C** | **δ^15^N** | **%C** | **%N** | **C:N** |
| --- | --- | --- | --- | --- | --- | --- | --- | --- | --- | --- | --- |
| AST01 | All Saints | 75 | Pre-Conquest | Sheep/Goat | Mandible | R | -20.6 | 6.9 | 38.2 | 13.6 | 3.3 |
| AST02 | All Saints | 75 | Pre-Conquest | Sheep/Goat | Mandible | R | -20.6 | 5.5 | 37.5 | 13.4 | 3.3 |
| AST03 | All Saints | 75 | Pre-Conquest | Sheep/Goat | Mandible | R | -21.0 | 7.2 | 38.1 | 13.7 | 3.2 |
| AST04 | All Saints | 75 | Pre-Conquest | Sheep/Goat | Mandible | R | -21.3 | 4.5 | 42.4 | 15.2 | 3.2 |
| AST05 | All Saints | 75 | Pre-Conquest | Sheep/Goat | Mandible | R | -22.0 | 8.0 | 41.5 | 14.9 | 3.2 |
| AST06 | All Saints | 75 | Pre-Conquest | Cattle | Mandible | R | -21.5 | 9.1 | 39.8 | 14.4 | 3.2 |
| AST07 | All Saints | 75 | Pre-Conquest | Cattle | Mandible | R | -21.9 | 8.1 | 40.5 | 14.7 | 3.2 |
| AST08 | All Saints | 75 | Pre-Conquest | Cattle | Mandible | R | -21.0 | 5.7 | 40.2 | 14.4 | 3.3 |
| AST09 | All Saints | 75 | Pre-Conquest | Cattle | Mandible | R | -21.5 | 7.4 | 40.0 | 14.3 | 3.3 |
| AST10 | All Saints | 94 | Pre-Conquest | Cattle | Mandible | R | -22.0 | 5.6 | 37.5 | 13.7 | 3.2 |
| AST11 | All Saints | 75 | Pre-Conquest | Pig | Tibia | L | -21.0 | 5.7 | 41.8 | 14.0 | 3.5 |
| AST12 | All Saints | 75 | Pre-Conquest | Pig | Tibia | L | -21.2 | 6.0 | 41.3 | 14.8 | 3.3 |
| AST13 | All Saints | 75 | Pre-Conquest | Pig | Tibia | L | -20.4 | 4.3 | 36.4 | 12.8 | 3.3 |
| AST14 | All Saints | 75 | Pre-Conquest | Pig | Tibia | R | -21.9 | 7.2 | 41.8 | 14.8 | 3.3 |
| AST15 | All Saints | 75 | Pre-Conquest | Pig | Tibia | L | -20.6 | 6.2 | 38.7 | 13.9 | 3.2 |
| OXC16 | Oxford Castle | 25 | Pre-Conquest | Pig | Tibia | R | -21.3 | 6.4 | 42.4 | 14.9 | 3.3 |
| OXC17 | Oxford Castle | 23 | Pre-Conquest | Pig | Maxilla | R | -21.7 | 10.9 | 34.2 | 12.1 | 3.3 |
| OXC18 | Oxford Castle | 30 | Pre-Conquest | Pig | Maxilla | R | -20.9 | 6.6 | 44.0 | 15.6 | 3.3 |
| OXC19 | Oxford Castle | 23 | Pre-Conquest | Pig | Pelvis | R | -21.3 | 6.2 | 41.7 | 15.1 | 3.2 |
| OXC20 | Oxford Castle | 28B | Pre-Conquest | Pig | Maxilla | L | -20.3 | 7.3 | 42.2 | 15.2 | 3.2 |
| OXC21 | Oxford Castle | 28B | Pre-Conquest | Sheep/Goat | Mandible | L | -21.7 | 10.7 | 23.4 | 8.3 | 3.3 |
| OXC22 | Oxford Castle | 25 | Pre-Conquest | Sheep/Goat | Mandible | R | -21.7 | 5.5 | 49.1 | 17.3 | 3.3 |
| OXC23 | Oxford Castle | 23 | Pre-Conquest | Sheep/Goat | Mandible | L | -21.7 | 7.3 | 42.2 | 14.7 | 3.3 |
| OXC24 | Oxford Castle | 23 | Pre-Conquest | Sheep/Goat | Mandible | L | -21.6 | 5.9 | 24.4 | 8.7 | 3.3 |
| OXC25 | Oxford Castle | 28B | Pre-Conquest | Sheep/Goat | Radius | L | -21.0 | 4.2 | 42.0 | 15.0 | 3.3 |
| OXC26 | Oxford Castle | 28B | Pre-Conquest | Cattle | Tibia | R | -22.3 | 6.5 | 41.3 | 14.5 | 3.3 |
| OXC27 | Oxford Castle | 28B | Pre-Conquest | Cattle | Tibia | L | -21.9 | 6.3 | 41.1 | 14.7 | 3.3 |
| OXC28 | Oxford Castle | 30 | Pre-Conquest | Cattle | Mandible | L | -21.6 | 6.1 | 41.9 | 15.0 | 3.3 |
| OXC29 | Oxford Castle | 28B | Pre-Conquest | Cattle | Mandible | R | -22.3 | 5.4 | 40.0 | 14.1 | 3.3 |
| OXC30 | Oxford Castle | 30 | Pre-Conquest | Cattle | Mandible | L | -21.6 | 6.3 | 40.4 | 14.6 | 3.2 |
| SAP31 | St Aldate's PS | 38 | Post-Conquest | Sheep/Goat | Tibia | L | -22.2 | 5.5 | 39.6 | 14.0 | 3.3 |
| SAP32 | St Aldate's PS | 38 | Post-Conquest | Sheep/Goat | Tibia | L | -22.2 | 7.1 | 37.9 | 13.2 | 3.3 |
| SAP33 | St Aldate's PS | 38 | Post-Conquest | Sheep/Goat | Tibia | L | -22.7 | 6.6 | 30.4 | 10.8 | 3.3 |
| SAP34 | St Aldate's PS | 38 | Post-Conquest | Sheep/Goat | Femur | R | -21.5 | 5.7 | 24.6 | 8.7 | 3.3 |
| SAP35 | St Aldate's PS | 38 | Post-Conquest | Sheep/Goat | Femur | R | -22.1 | 4.7 | 35.9 | 12.6 | 3.3 |
| SAP36 | St Aldate's PS | 38 | Post-Conquest | Pig | Ulna | L | -21.6 | 7.3 | 26.8 | 9.4 | 3.3 |
| SAP37 | St Aldate's PS | 38 | Post-Conquest | Pig | Humerus | L | -22.0 | 7.7 | 34.5 | 11.5 | 3.5 |
| SAP38 | St Aldate's PS | 38 | Post-Conquest | Pig | Humerus | R | -21.9 | 7.0 | 36.1 | 12.7 | 3.3 |
| SAP39 | St Aldate's PS | 38 | Post-Conquest | Pig | Femur | R | -21.0 | 9.2 | 40.2 | 14.1 | 3.3 |
| SAP40 | St Aldate's PS | 38 | Post-Conquest | Pig | Ulna | L | -21.6 | 6.5 | 34.4 | 12.2 | 3.3 |
| SAP41 | St Aldate's PS | 38 | Post-Conquest | Cattle | Tibia | R | -21.5 | 5.4 | 42.4 | 15.1 | 3.3 |
| SAP42 | St Aldate's PS | 42 | Post-Conquest | Cattle | Tibia | L | -21.6 | 9.5 | 38.3 | 13.6 | 3.3 |
| SAP43 | St Aldate's PS | 38 | Post-Conquest | Cattle | Tibia | L | -21.8 | 8.6 | 38.2 | 13.6 | 3.3 |
| SAP44 | St Aldate's PS | 38 | Post-Conquest | Cattle | Radius | L | -21.6 | 6.4 | 29.9 | 10.7 | 3.3 |
| SAP45 | St Aldate's PS | 38 | Post-Conquest | Cattle | Radius | L | -21.9 | 8.5 | 39.5 | 13.8 | 3.3 |
| SAL46 | St Aldate's LAPS | 204 | Post-Conquest | Sheep/Goat | Tibia | L | -20.8 | 7.7 | 36.9 | 13.0 | 3.3 |
| SAL47 | St Aldate's LAPS | 154 | Post-Conquest | Sheep/Goat | Tibia | L | -22.3 | 10.8 | 41.7 | 14.8 | 3.3 |
| SAL48 | St Aldate's LAPS | 204 | Post-Conquest | Sheep/Goat | Tibia | L | -22.0 | 4.9 | 40.7 | 14.3 | 3.3 |
| SAL49 | St Aldate's LAPS | 204 | Post-Conquest | Sheep/Goat | Tibia | L | -22.3 | 4.5 | 38.7 | 13.8 | 3.3 |
| SAL50 | St Aldate's LAPS | 153 | Post-Conquest | Sheep/Goat | Tibia | L | -22.3 | 8.3 | 35.8 | 12.8 | 3.3 |
| SAL51 | St Aldate's LAPS | 153 | Post-Conquest | Cattle | Mandible | R | -22.0 | 8.7 | 40.6 | 14.7 | 3.2 |
| SAL52 | St Aldate's LAPS | 153 | Post-Conquest | Cattle | Mandible | R | -22.1 | 5.1 | 36.3 | 12.6 | 3.4 |
| SAL53 | St Aldate's LAPS | 204 | Post-Conquest | Cattle | Radius | R | -21.8 | 3.2 | 41.7 | 15.0 | 3.2 |
| SAL54 | St Aldate's LAPS | 153 | Post-Conquest | Cattle | Radius | R | -22.0 | 6.3 | 40.7 | 14.5 | 3.3 |
| SAL55 | St Aldate's LAPS | 156 | Post-Conquest | Cattle | Phalanx I | - | -22.4 | 7.8 | 41.5 | 15.0 | 3.2 |
| SAL56 | St Aldate's LAPS | 153 | Post-Conquest | Pig | Mandible | R | -21.3 | 8.3 | 35.2 | 11.8 | 3.5 |
| SAL57 | St Aldate's LAPS | 153 | Post-Conquest | Pig | Metacarpal 4 | L | -21.2 | 8.3 | 34.4 | 12.2 | 3.3 |
| SAL58 | St Aldate's LAPS | 153 | Post-Conquest | Pig | Premaxilla | L | -21.2 | 6.8 | 30.4 | 10.7 | 3.3 |
| SAP59 | St Aldate's PS | 38 | Post-Conquest | Pig | Mandible | L | -21.9 | 8.1 | 29.6 | 10.4 | 3.3 |
| SAP60 | St Aldate's LAPS | 42 | Post-Conquest | Pig | Calcaneum | L | -22.0 | 7.9 | 34.4 | 12.2 | 3.3 |
