# Supplementary material for: The dietary impact of the Norman Conquest: A multiproxy archaeological investigation of Oxford, UK
Source: PLoS One. 2020 Jul 6;15(7):e0235005. doi: 10.1371/journal.pone.0235005 (PMC7337355; doi:10.1371/journal.pone.0235005)
Supplement: S3 Table — (DOCX) [file pone.0235005.s004.docx]

**S4 Table. Sample details and stable isotope value results for human remains.**

| **Sample** | **Site** | **Burial/**  **Context** | **Date** | **Element** | **Side** | **Sex** | **Age (years)** | **δ^13^C** | **δ^15^N** | **%C** | **%N** | **C:N** |
| --- | --- | --- | --- | --- | --- | --- | --- | --- | --- | --- | --- | --- |
| AST61 | All Saints | 61 | 11th Century | Rib | R | F | 18-25 | -19.4 | 11.4 | 33.0 | 12.1 | 3.2 |
| AST62 | All Saints | 63 | 11th Century | Rib | R | M | 40+ | -19.6 | 12.3 | 31.8 | 11.6 | 3.2 |
| AST63 | All Saints | 66 | 11th Century | Rib | L | M | 25-34 | -19.6 | 10.7 | 39.2 | 14.2 | 3.2 |
| AST64 | All Saints | 55 | 11th Century | Rib | R | F | 25+ | -19.3 | 11.6 | 40.9 | 15.0 | 3.2 |
| AST65 | All Saints | 58/2 | Post-Conquest | Rib | R | M | 45+ | -19.2 | 12.5 | 26.4 | 9.4 | 3.3 |
| AST66 | All Saints | 54 | 11th Century | Rib | R | F | adult | -19.5 | 11.4 | 27.2 | 9.9 | 3.2 |
| AST67 | All Saints | 57 | 11th Century | Rib | L | M | 35+ | -19.6 | 11.7 | 39.4 | 14.3 | 3.2 |
| AST68 | All Saints | 53 | 11th Century | Rib | R | M | 17-25 | -19.6 | 11.7 | 24.2 | 8.4 | 3.3 |
| AST69 | All Saints | 50 | Post-Conquest | Rib | L | U | 12-14 | -18.8 | 12.7 | 40.6 | 14.8 | 3.2 |
| AST70 | All Saints | 64 | 11th Century | Tibia | L | F | 20-23 | -19.6 | 10.9 | 29.6 | 10.5 | 3.3 |
| AST71 | All Saints | 65 | Post-Conquest | Femur | R | U | 20-25 | -19.5 | 11.1 | 36.2 | 13.1 | 3.2 |
| AST72 | All Saints | 69 | 11th Century | Tibia | L | U | 16-23 | -19.5 | 11.1 | 38.6 | 14.1 | 3.2 |
| WTG73 |  | ctx 5 | Post-Conquest | Femur | R | U |  | -19.8 | 11.2 | 36.5 | 13.1 | 3.2 |
| WTG74 | Westgate | ctx 5 | Post-Conquest | Femur | R | U |  | -19.4 | 10.9 | 39.2 | 14.0 | 3.3 |
| WTG75 | Westgate | ctx 5 | Post-Conquest | Femur | R | U |  | -19.6 | 10.1 | 45.0 | 16.3 | 3.2 |
| WTG76 | Westgate | ctx 5 | Post-Conquest | Femur | R | U |  | -19.8 | 11.4 | 19.3 | 6.9 | 3.3 |
| WTG77 | Westgate | ctx 5 | Post-Conquest | Femur | R | U |  | -19.5 | 10.2 | 41.4 | 15.0 | 3.2 |
| WTG78 | Westgate | ctx 5 | Post-Conquest | Femur | R | U |  | -19.7 | 12.4 | 42.8 | 15.4 | 3.2 |
| WTG79 | Westgate | ctx 5 | Post-Conquest | Femur | R | U |  | -19.6 | 10.2 | 35.2 | 12.7 | 3.2 |
| WTG80 | Westgate | ctx 5 | Post-Conquest | Femur | R | U |  | -19.4 | 12.1 | 41.6 | 15.0 | 3.2 |
| WTG81 | Westgate | ctx 5 | Post-Conquest | Femur | R | U |  | -19.5 | 11.6 | 36.3 | 12.8 | 3.3 |
| WTG82 | Westgate | ctx 5 | Post-Conquest | Femur | R | U |  | -19.7 | 10.4 | 37.4 | 13.6 | 3.2 |
| WTG83 | Westgate | ctx 5 | Post-Conquest | Femur | R | U |  | -19.2 | 10.5 | 40.0 | 14.3 | 3.3 |
| WTG84 | Westgate | ctx 5 | Post-Conquest | Femur | R | U |  | -19.2 | 10.0 | 37.8 | 13.7 | 3.2 |
| WTG85 | Westgate | ctx 5 | Post-Conquest | Femur | R | U |  | -19.3 | 10.2 | 36.2 | 13.0 | 3.2 |
| CCH87 | Christ Church | 418 | Pre-Conquest | Humerus | R | M | Adult | -19.6 | 9.4 | 40.5 | 14.6 | 3.2 |
| CCH88 | Christ Church | 427 | Pre-Conquest | Clavicle | L | U | Adult | -20.2 | 10.8 | 36.1 | 12.9 | 3.3 |
| CCH89 | Christ Church | 402 | Pre-Conquest | Femur | L | F | 45+ | -20.1 | 11.4 | 38.5 | 13.6 | 3.3 |
| CCH90 | Christ Church | 227 | Pre-Conquest | Femur | L | M | 25-40 | -19.8 | 10.9 | 35.0 | 12.4 | 3.3 |
| CCH91 | Christ Church | 262 | Pre-Conquest | Femur | R | M | 40+ | -18.9 | 11.8 | 37.3 | 13.4 | 3.2 |
| CCH92 | Christ Church | 289 | Pre-Conquest | Femur | L | M | 25-40 | -19.5 | 8.6 | 31.7 | 11.3 | 3.3 |
| OXM86 | Oxford Castle mound | III 8 | Pre-Conquest | Humerus | R | U |  | -19.6 | 12.4 | 32.5 | 11.6 | 3.3 |
| OXC99 | Oxford Castle | 5793 | 11th Century | Rib | R | F | 18-25 | -20.4 | 11.9 | 17.4 | 5.8 | 3.5 |
| OXC100 | Oxford Castle | 5804 | 11th Century | Rib | R | F | 18-25 | -19.7 | 11.4 | 35.5 | 12.2 | 3.4 |

Side: R=right, L=left; Sex: M=male, F=female, U=unsexed
