# Supplementary material for: The dietary impact of the Norman Conquest: A multiproxy archaeological investigation of Oxford, UK
Source: PLoS One. 2020 Jul 6;15(7):e0235005. doi: 10.1371/journal.pone.0235005 (PMC7337355; doi:10.1371/journal.pone.0235005)
Supplement: S4 Table — a. Data from bone collagen failed to meet quality control criteria. (DOCX) [file pone.0235005.s005.docx]

**S5 Table. Summary data from the nine incrementally-sampled second molars from All Saints (AST) and Oxford Castle (OXC).** a. Data from bone collagen failed to meet quality control criteria.

| **Context Number** | **Sample** | **Date** | **Age (years)** | **Sex** | **Increments (n)** | **Increment Range** | | **Increment Average** | | **Increment 1SD** | | **Bone Collagen Value** | |
| --- | --- | --- | --- | --- | --- | --- | --- | --- | --- | --- | --- | --- | --- |
|  |  |  |  |  |  | **δ^15^N ‰** | **δ^13^C ‰** | **δ^15^N ‰** | **δ^13^C ‰** | **δ^15^N ‰** | **δ^13^C ‰** | **δ^15^N ‰** | **δ^13^C ‰** |
| All Saints 63 | AST62 | 11^th^ Century | 40+ | M | 21 | 10.7 to 13.8 | -20.3 to -19.8 | 12.4 | -20.0 | 1.0 | 0.2 | 12.3 | -19.6 |
| All Saints 66 | AST63 | 11^th^ Century | 25-34 | M | 16 | 9.4 to 11.2 | -20.8 to -19.8 | 10.1 | -20.2 | 0.5 | 0.2 | 10.7 | -19.6 |
| All Saints 55 | AST64 | 11^th^ Century | 25+ | F | 17 | 10.7 to 11.8 | -20.4 to -19.5 | 11.3 | -19.9 | 0.3 | 0.3 | 11.6 | -19.3 |
| All Saints 58/2 | AST65 | Post-Conquest | 35+ | M | 16 | 9.9 to 12.4 | -20.4 to -19.2 | 11.3 | -19.6 | 0.8 | 0.3 | 12.5 | -19.2 |
| All Saints 53 | AST68 | 11^th^ Century | 17-25 | M | 10 | 11.3 to 12.1 | -20.3 to -19.5 | 11.6 | -19.8 | 0.3 | 0.3 | 11.7 | -19.6 |
| Oxford Castle 5793 | OXC99 | 11^th^ Century | 25-35 | F | 12 | 11.6 to 12.7 | -21.1 to -19.7 | 12.1 | -20.1 | 0.4 | 0.4 | 11.9 | -20.4 |
| Oxford Castle 5804 | OXC100 | 11^th^ Century | 25-35 | F | 15 | 10.6 to 12.0 | -20.5 to -19.5 | 11.5 | -19.9 | 0.3 | 0.3 | 11.4 | -19.7 |
| Oxford Castle 4240 | OXC101 | 11^th^ Century | 18-25 | F | 12 | 5.5 to 11.0 | -21.0 to -19.7 | 8.9 | -20.3 | 1.5 | 0.3 | -^a^ | -^a^ |
| Oxford Castle 5787 | OXC102 | 11^th^ Century | 10-12 | U | 13 | 7.8 to 11.0 | -20.2 to -19.2 | 9.7 | -19.7 | 1.0 | 0.3 | -^a^ | -^a^ |

Side: R=right, L=left; Sex: M=male, F=female, U=unsexed.
