# Supplementary material for: The dietary impact of the Norman Conquest: A multiproxy archaeological investigation of Oxford, UK
Source: PLoS One. 2020 Jul 6;15(7):e0235005. doi: 10.1371/journal.pone.0235005 (PMC7337355; doi:10.1371/journal.pone.0235005)
Supplement: S5 Table — (DOCX) [file pone.0235005.s006.docx]

**S6 Table. Full incremental δ^13^C and δ^15^N isotope data.**

| **Context/Skeleton No** | **Sample no.** | **Approx. Age at Increment** | **δ^13^C (‰)** | **δ^15^N (‰)** | **%C** | **%N** | **C:N** | **Repeats^b^** |
| --- | --- | --- | --- | --- | --- | --- | --- | --- |
| All Saints 63 | AST62.01 | 2.5 | -20.2 | 10.7 | 40.7 | 14.5 | 3.3 | 2 |
|  | AST62.02 | 3.1 | -20.3 | 13.6 | 40.7 | 13.9 | 3.4 | 2 |
|  | AST62.03 | 3.7 | -20.1 | 13.8 | 42.2 | 14.2 | 3.5 | 2 |
|  | AST62.04 | 4.3 | -19.8 | 13.8 | 41.5 | 13.8 | 3.5 | 1 |
|  | AST62.05 | 4.9 | -19.8 | 13.8 | 41.0 | 14.0 | 3.4 | 2 |
|  | AST62.06 | 5.5 | -20.3 | 13.4 | 42.5 | 14.0 | 3.5 | 2 |
|  | AST62.07 | 6.1 | -20.1 | 13.3 | 41.7 | 14.2 | 3.4 | 2 |
|  | AST62.08 | 6.7 | -20.0 | 13.1 | 42.5 | 14.6 | 3.4 | 2 |
|  | AST62.09 | 7.3 | -19.8 | 12.6 | 42.9 | 14.7 | 3.4 | 2 |
|  | AST62.10 | 7.9 | -20.0 | 12.0 | 43.3 | 14.6 | 3.5 | 2 |
|  | AST62.11 | 8.5 | -20.1 | 11.0 | 40.4 | 14.2 | 3.3 | 2 |
|  | AST62.12 | 9.1 | -20.0 | 11.8 | 44.6 | 15.7 | 3.3 | 2 |
|  | AST62.13 | 9.7 | -20.0 | 12.3 | 46.1 | 16.2 | 3.3 | 2 |
|  | AST62.14 | 10.3 | -20.0 | 12.2 | 43.0 | 15.1 | 3.3 | 2 |
|  | AST62.15 | 10.9 | -19.8 | 12.3 | 42.3 | 15.0 | 3.3 | 2 |
|  | AST62.16 | 11.5 | -19.9 | 11.9 | 42.2 | 14.9 | 3.3 | 2 |
|  | AST62.17 | 12.1 | -20.0 | 11.9 | 41.7 | 14.6 | 3.3 | 2 |
|  | AST62.18 | 12.7 | -19.8 | 11.8 | 42.6 | 15.1 | 3.3 | 2 |
|  | AST62.19 | 13.3 | -20.0 | 11.4 | 42.1 | 14.7 | 3.4 | 2 |
|  | AST62.20 | 13.9 | -20.2 | 11.0 | 41.9 | 14.6 | 3.3 | 2 |
|  | AST62.21 | 14.5 | -20.1 | 12.3 | 44.0 | 15.7 | 3.3 | 2 |
| All Saints 66 | AST63.01 | 2.5 | -20.1 | 10.6 | 41.3 | 13.9 | 3.4 | 2 |
|  | AST63.02 | 3.3 | -20.2 | 9.8 | 39.8 | 13.8 | 3.4 | 2 |
|  | AST63.03 | 4.1 | -20.5 | 9.4 | 38.5 | 12.9 | 3.5 | 2 |
|  | AST63.04 | 4.9 | -20.1 | 9.6 | 39.0 | 13.7 | 3.3 | 2 |
|  | AST63.05 | 5.7 | -20.1 | 9.7 | 38.9 | 13.4 | 3.4 | 2 |
|  | AST63.06 | 6.5 | -19.8 | 10.1 | 39.2 | 13.8 | 3.3 | 2 |
|  | AST63.07 | 7.3 | -20.1 | 10.7 | 36.5 | 12.7 | 3.4 | 2 |
|  | AST63.08 | 8.1 | -20.1 | 10.4 | 38.7 | 13.4 | 3.4 | 2 |
|  | AST63.09 | 8.9 | -20.3 | 10.5 | 40.2 | 14.0 | 3.4 | 2 |
|  | AST63.10 | 9.7 | -20.0 | 11.2 | 43.0 | 15.1 | 3.3 | 3 |
|  | AST63.11 | 10.5 | -20.5 | 10.4 | 37.9 | 12.9 | 3.4 | 2 |
|  | AST63.12 | 11.3 | -20.8 | 10.2 | 41.0 | 13.3 | 3.6 | 2 |
|  | AST63.13 | 12.1 | -20.2 | 9.6 | 39.4 | 13.6 | 3.4 | 2 |
|  | AST63.14 | 12.9 | -20.2 | 9.5 | 39.0 | 13.6 | 3.3 | 2 |
|  | AST63.15 | 13.7 | -20.1 | 9.9 | 37.7 | 13.1 | 3.4 | 2 |
|  | AST63.16 | 14.5 | -20.4 | 10.1 | 38.0 | 12.8 | 3.5 | 2 |
| All Saints 55 | AST64.01 | 2.5 | -19.9 | 11.4 | 27.5 | 9.4 | 3.4 | 2 |
|  | AST64.02 | 3.3 | -19.7 | 11.6 | 34.8 | 12.0 | 3.4 | 2 |
|  | AST64.03 | 4.1 |  |  |  |  |  |  |
|  | AST64.04 | 4.9 | -20.4 | 11.5 | 23.3 | 7.5 | 3.6 | 1 |
|  | AST64.05 | 5.7 | -19.7 | 11.5 | 38.8 | 13.7 | 3.3 | 2 |
|  | AST64.06 | 6.5 | -19.8 | 11.5 | 33.8 | 12.1 | 3.3 | 1 |
|  | AST64.07 | 7.3 | -19.8 | 11.8 | 37.7 | 13.4 | 3.3 | 2 |
|  | AST64.08 | 8.1 | -19.5 | 11.7 | 38.4 | 13.7 | 3.3 | 2 |
|  | AST64.09 | 8.9 | -20.0 | 11.5 | 39.5 | 13.6 | 3.4 | 2 |
|  | AST64.10 | 9.7 | -19.6 | 11.5 | 27.6 | 9.8 | 3.3 | 2 |
|  | AST64.11 | 10.5 | -19.6 | 11.3 | 37.5 | 13.2 | 3.3 | 2 |
|  | AST64.12 | 11.3 | -19.8 | 11.0 | 41.1 | 14.5 | 3.3 | 2 |
|  | AST64.13 | 12.1 | -20.1 | 10.7 | 39.1 | 14.0 | 3.3 | 2 |
|  | AST64.14 | 12.9 | -20.2 | 10.7 | 40.6 | 14.4 | 3.3 | 2 |
|  | AST64.15 | 13.7 | -20.0 | 10.9 | 40.2 | 14.4 | 3.3 | 2 |
|  | AST64.16 | 14.5 | -20.3 | 11.2 | 43.3 | 15.3 | 3.3 | 2 |
|  | AST64.17 | 15.3 | -20.2 | 11.0 | 41.2 | 14.7 | 3.3 | 2 |
| All Saints 58/2 | AST65.01 | 2.5 | -19.7 | 12.4 | 41.4 | 14.3 | 3.4 | 2 |
|  | AST65.02 | 3.3 | -20.4 | 12.0 | 45.2 | 14.8 | 3.6 | 2 |
|  | AST65.03 | 4.1 | -20.1 | 11.5 | 45.3 | 16.1 | 3.3 | 2 |
|  | AST65.04 | 4.9 | -19.7 | 12.1 | 41.5 | 14.6 | 3.4 | 2 |
|  | AST65.05 | 5.7 | -20.0 | 12.4 | 43.8 | 14.5 | 3.5 | 2 |
|  | AST65.06 | 6.5 | -19.4 | 12.0 | 43.6 | 15.2 | 3.4 | 2 |
|  | AST65.07 | 7.3 | -19.3 | 11.7 | 45.2 | 15.8 | 3.3 | 2 |
|  | AST65.08 | 8.1 | -19.2 | 11.2 | 41.8 | 14.5 | 3.4 | 2 |
|  | AST65.09 | 8.9 | -19.3 | 10.6 | 44.4 | 15.5 | 3.3 | 2 |
|  | AST65.10 | 9.7 | -19.3 | 10.3 | 44.2 | 15.6 | 3.3 | 2 |
|  | AST65.11 | 10.5 | -19.4 | 9.9 | 44.9 | 15.9 | 3.3 | 2 |
|  | AST65.12 | 11.3 | -19.5 | 10.1 | 42.6 | 15.1 | 3.3 | 2 |
|  | AST65.13 | 12.1 | -19.5 | 11.2 | 43.8 | 15.5 | 3.3 | 2 |
|  | AST65.14 | 12.9 | -19.7 | 11.5 | 40.3 | 14.3 | 3.3 | 2 |
|  | AST65.15 | 13.7 | -20.0 | 11.3 | 42.8 | 15.0 | 3.3 | 2 |
|  | AST65.16 | 14.5 | -20.0 | 10.7 | 43.4 | 15.4 | 3.3 | 2 |
| All Saints 53 | AST68.01 | 2.5 | -20.3 | 11.9 | 36.1 | 12.1 | 3.5 | 1 |
|  | AST68.02 | 3.8 | -19.6 | 11.9 | 43.1 | 15.2 | 3.3 | 1 |
|  | AST68.03 | 5.1 | -19.5 | 11.7 | 41.5 | 14.6 | 3.3 | 2 |
|  | AST68.04 | 6.4 | -19.6 | 11.5 | 39.5 | 13.7 | 3.4 | 2 |
|  | AST68.05 | 7.7 | -19.9 | 11.5 | 41.4 | 14.0 | 3.4 | 2 |
|  | AST68.06 | 9 | -20.2 | 11.3 | 38.5 | 12.9 | 3.5 | 2 |
|  | AST68.07 | 10.3 | -19.6 | 11.4 | 39.6 | 14.0 | 3.3 | 2 |
|  | AST68.08 | 11.6 | -19.9 | 11.4 | 39.0 | 13.6 | 3.3 | 2 |
|  | AST68.09 | 12.9 | -20.1 | 11.6 | 40.3 | 13.2 | 3.5 | 2 |
|  | AST68.10 | 13.2 | -19.6 | 12.1 | 41.1 | 14.7 | 3.3 | 2 |
| Oxford Castle 5793 | OXC 99.01 | 2.9 | -20.2 | 11.6 | 30.0 | 10.4 | 3.4 | 2 |
|  | OXC 99.02 | 3.7 | -19.8 | 11.7 | 36.6 | 12.5 | 3.4 | 2 |
|  | OXC 99.03 | 4.5 | -20.0 | 11.8 | 34.8 | 12.2 | 3.3 | 2 |
|  | OXC 99.04 | 5.7 | -19.9 | 11.8 | 34.7 | 11.9 | 3.4 | 2 |
|  | OXC 99.05 | 7.3 | -19.7 | 12.2 | 36.7 | 13.0 | 3.3 | 2 |
|  | OXC 99.06 | 8.1 | -20.0 | 12.5 | 36.4 | 12.5 | 3.4 | 2 |
|  | OXC 99.07 | 8.9 | -20.0 | 12.5 | 37.4 | 13.1 | 3.3 | 2 |
|  | OXC 99.08 | 9.7 | -19.8 | 12.7 | 36.5 | 12.6 | 3.4 | 1 |
|  | OXC 99.09 | 10.5 | -20.2 | 11.9 | 36.3 | 12.6 | 3.4 | 2 |
|  | OXC 99.10 | 11.3 | - | - | - | - |  |  |
|  | OXC 99.11 | 12.1 | -20.0 | 12.4 | 35.2 | 12.3 | 3.3 | 2 |
|  | OXC 99.12 | 12.9 | -21.1 | 12.2 | 33.1 | 11.4 | 3.4 | 2 |
|  | OXC 99.13 | 13.7 | - | - | - | - |  |  |
|  | OXC 99.14 | 14.5 | -20.0 | 12.0 | 37.0 | 12.8 | 3.4 | 2 |
| Oxford Castle 5804 | OXC 100.01 | 3.7 | -20.1 | 11.7 | 38.4 | 13.5 | 3.3 | 2 |
|  | OXC 100.02 | 4.9 | -20.5 | 11.3 | 45.1 | 15.5 | 3.4 | 2 |
|  | OXC 100.03 | 5.5 | -20.0 | 11.3 | 38.4 | 13.9 | 3.2 | 2 |
|  | OXC 100.04 | 6.4 | -19.7 | 11.4 | 38.4 | 13.8 | 3.3 | 2 |
|  | OXC 100.05 | 7 | - | - | - | - |  |  |
|  | OXC 100.06 | 8.2 | -19.8 | 11.8 | 42.0 | 15.1 | 3.3 | 2 |
|  | OXC 100.07 | 8.8 | -20.0 | 11.5 | 38.4 | 13.7 | 3.3 | 2 |
|  | OXC 100.08 | 9.4 | -19.9 | 11.4 | 41.2 | 14.7 | 3.3 | 2 |
|  | OXC 100.09 | 10.6 | -20.0 | 11.6 | 41.2 | 14.6 | 3.3 | 2 |
|  | OXC 100.10 | 11.2 | -19.8 | 11.5 | 40.4 | 14.4 | 3.3 | 2 |
|  | OXC 100.11 | 11.8 | -19.5 | 11.5 | 38.8 | 14.0 | 3.2 | 2 |
|  | OXC 100.12 | 12.4 | -19.5 | 11.7 | 38.7 | 14.0 | 3.2 | 2 |
|  | OXC 100.13 | 13 | -19.6 | 12.0 | 42.5 | 15.2 | 3.3 | 2 |
|  | OXC 100.14 | 13.6 | -19.8 | 12.0 | 38.0 | 13.2 | 3.4 | 1 |
|  | OXC 100.15 | 14.2 | -20.0 | 10.6 | 33.5 | 11.7 | 3.4 | 1 |
|  | OXC 100.16 | 14.8 | -20.0 | 11.6 | 36.8 | 12.7 | 3.4 | 1 |
| Oxford Castle 4240 | OXC 101.01 | 2.9 | -20.4 | 10.5 | 36.9 | 13.2 | 3.3 | 2 |
|  | OXC 101.02 | 3.6 | -20.5 | 9.9 | 34.0 | 12.1 | 3.3 | 2 |
|  | OXC 101.03 | 4.3 | -20.4 | 8.6 | 33.9 | 12.2 | 3.3 | 2 |
|  | OXC 101.04 | 5.4 | -21.0 | 9.1 | 36.8 | 12.5 | 3.4 | 2 |
|  | OXC 101.05 | 6.1 | -19.7 | 11.0 | 36.8 | 13.1 | 3.3 | 2 |
|  | OXC 101.06 | 7.5 | -20.1 | 9.0 | 33.3 | 12.1 | 3.2 | 2 |
|  | OXC 101.07 | 8.2 | -20.3 | 8.9 | 37.8 | 13.4 | 3.3 | 2 |
|  | OXC 101.08 | 9.6 | -20.1 | 8.9 | 37.0 | 13.3 | 3.3 | 2 |
|  | OXC 101.09 | 11 | -20.4 | 6.8 | 38.2 | 14.0 | 3.2 | 2 |
|  | OXC 101.10 | 11.7 | -20.1 | 8.7 | 35.3 | 12.8 | 3.2 | 2 |
|  | OXC 101.11 | 13.1 | -20.0 | 9.5 | 26.2 | 9.3 | 3.3 | 2 |
|  | OXC 101.12 | 13.8 | -20.2 | 5.5 | 20.6 | 7.7 | 3.1 | 1 |
|  | OXC 101.13 | 14.5 | - | - | - | - |  |  |
| Oxford Castle 5787 | OXC 102.01 | 2.5 | -19.9 | 10.3 | 32.4 | 11.3 | 3.4 | 2 |
|  | OXC 102.02 | 3.2 | -19.7 | 10.5 | 33.7 | 12.0 | 3.3 | 2 |
|  | OXC 102.03 | 3.9 | -19.7 | 10.5 | 30.3 | 10.6 | 3.3 | 2 |
|  | OXC 102.04 | 4.6 | -20.2 | 10.8 | 37.4 | 12.7 | 3.4 | 2 |
|  | OXC 102.05 | 5.3 | -19.8 | 11.0 | 35.9 | 12.6 | 3.3 | 2 |
|  | OXC 102.06 | 6 | -19.7 | 9.5 | 37.6 | 13.6 | 3.2 | 2 |
|  | OXC 102.07 | 6.7 | -19.6 | 9.8 | 35.9 | 12.7 | 3.3 | 2 |
|  | OXC 102.08 | 7.4 | -19.2 | 9.8 | 35.3 | 12.6 | 3.3 | 2 |
|  | OXC 102.09 | 8.1 | -19.4 | 10.0 | 36.9 | 13.0 | 3.3 | 2 |
|  | OXC 102.10 | 8.9 | -19.2 | 9.0 | 37.6 | 11.8 | 3.2 | 2 |
|  | OXC 102.11 | 9.6 | -19.4 | 8.9 | 31.3 | 11.0 | 3.3 | 2 |
|  | OXC 102.12 | 10.3 | -20.0 | 7.8 | 31.6 | 10.6 | 3.5 | 2 |
|  | OXC 102.13 | 11 | -20.2 | 8.4 | 25.0 | 8.3 | 3.5 | 1 |
|  | OXC 102.14 | 11.7 | - | - | - | - |  |  |

Samples with absent data produced too little collagen for a single analysis. a. Some samples yielded insufficient collagen for replicate analysis.
