# Supplementary material for: The dietary impact of the Norman Conquest: A multiproxy archaeological investigation of Oxford, UK
Source: PLoS One. 2020 Jul 6;15(7):e0235005. doi: 10.1371/journal.pone.0235005 (PMC7337355; doi:10.1371/journal.pone.0235005)
Supplement: S6 Table — (DOCX) [file pone.0235005.s007.docx]

**S7 Table. Prevalence of calculus and abscesses by period.**

**S7(A) Calculus**

|  | **Pre-Conquest** | **Post-Conquest** | ***Total*** |
| --- | --- | --- | --- |
| **Absent** | 48 | 136 | *148* |
| **Present** | 11 | 40 | *51* |
| ***Total*** | *59* | *176* | *235* |

**S7(B) Abscesses**

|  | **Pre-Conquest** | **Post-Conquest** | ***Total*** |
| --- | --- | --- | --- |
| **Absent** | 53 | 167 | *220* |
| **Present** | 6 | 9 | *15* |
| ***Total*** | *59* | *176* | *235* |
